# Sex differences in airway disease: estrogen and airway surface liquid dynamics

**DOI:** 10.1186/s13293-024-00633-z

**Published:** 2024-07-18

**Authors:** Brian J. Harvey, Noel G. McElvaney

**Affiliations:** 1 Faculty of Medicine and Health Sciences, Royal College of Surgeons in Ireland, 126 St Stephens Green, Dublin 2, Ireland; 2 Department of Medicine, RCSI ERC, Beaumont Hospital, Dublin 2, Ireland

**Keywords:** Estrogen, Cystic fibrosis, Asthma, COVID-19, Ion channels, Airway surface liquid

## Abstract

Biological sex differences exist for many airway diseases in which females have either worse or better health outcomes. Inflammatory airway diseases such as cystic fibrosis (CF) and asthma display a clear male advantage in post-puberty while a female benefit is observed in asthma during the pre-puberty years. The influence of menstrual cycle stage and pregnancy on the frequency and severity of pulmonary exacerbations in CF and asthma point to a role for sex steroid hormones, particularly estrogen, in underpinning biological sex differences in these diseases. There are many ways by which estrogen may aggravate asthma and CF involving disturbances in airway surface liquid (ASL) dynamics, inappropriate hyper-immune and allergenic responses, as well as exacerbation of pathogen virulence. The deleterious effect of estrogen on pulmonary function in CF and asthma contrasts with the female advantage observed in airway diseases characterised by pulmonary edema such as pneumonia, acute respiratory distress syndrome (ARDS) and COVID-19. Airway surface liquid hypersecretion and alveolar flooding are hallmarks of ARDS and COVID-19, and contribute to the morbidity and mortality of severe forms of these diseases. ASL dynamics encompasses the intrinsic features of the thin lining of fluid covering the airway epithelium which regulate mucociliary clearance (ciliary beat, ASL height, volume, pH, viscosity, mucins, and channel activating proteases) in addition to innate defence mechanisms (pathogen virulence, cytokines, defensins, specialised pro-resolution lipid mediators, and metabolism). Estrogen regulation of ASL dynamics contributing to biological sex differences in CF, asthma and COVID-19 is a major focus of this review.

**Graphical Abstract:**

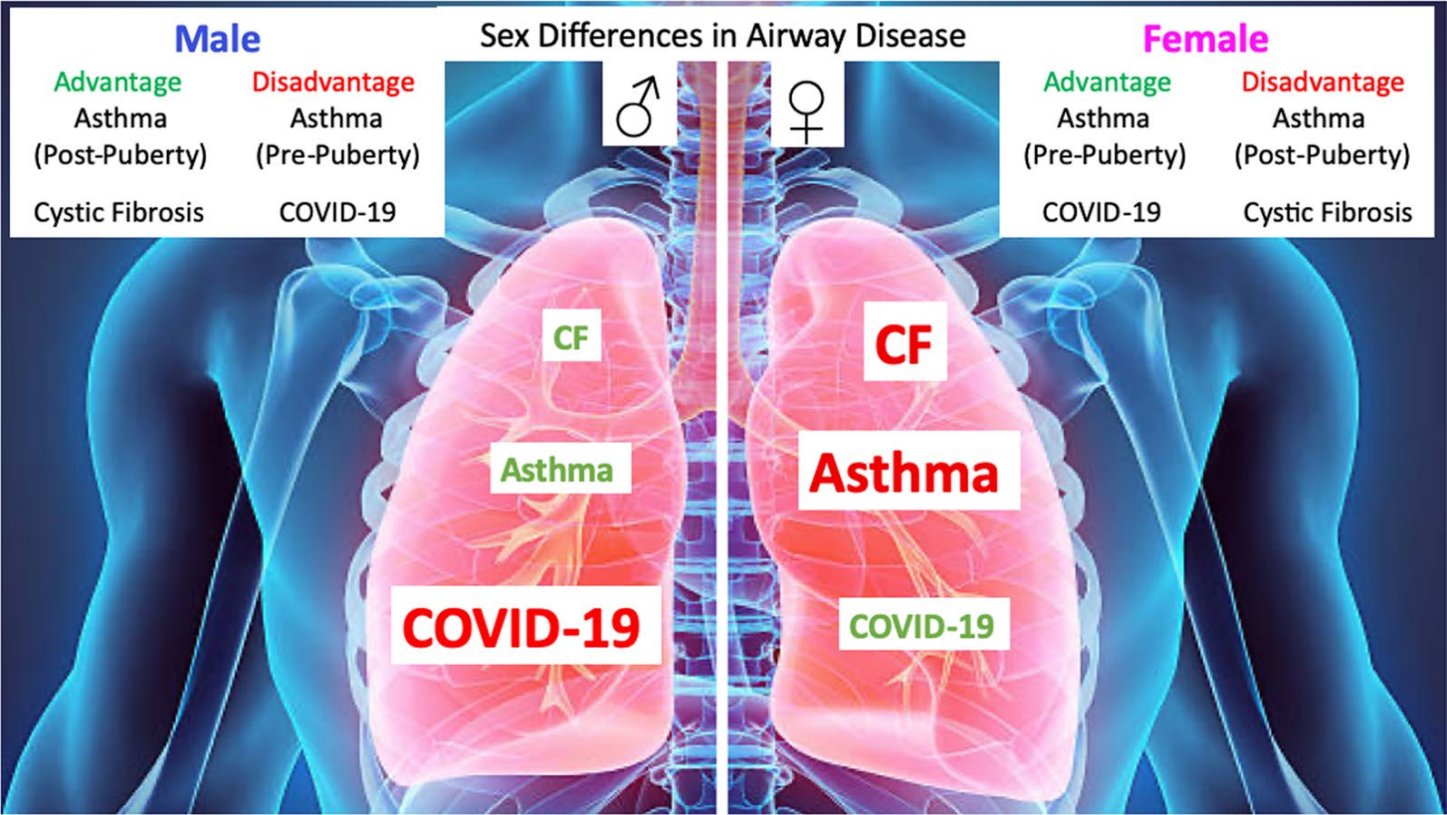

## Plain Language Summary

Sex differences exist in many airway diseases in which females have either worse or better health outcomes. These include cystic fibrosis (CF) and asthma where females post-puberty have more frequent lung exacerbations and higher mortality. Lung infections and difficulty in breathing become worse in post-puberty in females and during the ovulation period of the menstrual cycle, and in pregnancy, indicating a role for sex hormones in underpinning sex differences in CF and asthma. Evidence also exists for sex differences with a female advantage in airway diseases which are characterised by flooding of the airways, as in pneumonia and COVID-19, where females have better lung function and lower risk of death than males. The female sex hormone estrogen plays an important role in determining the role of sex biology in airway disease severity and mortality. Estrogen can control the movement of salt and water in and out of the lung airway tubes and dehydrate the lung surface to make it more sticky with mucus, as observed in CF and asthma, thus worsening the condition. In contrast, estrogen can have beneficial effects in lowering the volume of water in the lungs in COVID-19 thus alleviating flooding of the airways. This review focusses on the biology of sex differences in CF, asthma and COVID-19, and the cellular mechanisms by which estrogen can have either detrimental or beneficial effects in these diseases.

## Highlights

- Airway diseases such as cystic fibrosis (CF), asthma and COVID-19 show marked sex differences in their morbidity and mortality
- Estrogen and female sex are associated with exacerbated CF and asthma
- Estrogen aggravates CF and asthma by compromising the optimal airway surface liquid dynamics for effective mucociliary clearance and by exacerbating bacterial virulence
- Estrogen confers a female advantage in COVID-19 by reducing airway flooding and reinforcing immunity.

## Background

Sex differences exist in the biology of airway diseases between males and females [1, 2]. An apparent sex difference and ‘gender gap’ have been observed in the incidence and detection of various airway diseases [3, 4]. Here we define sex differences as comprising biological variations including physiology and genetics. Gender on the other hand is a social construct and concerns differences *inter alia* in socio-economic status, education, health care access and behavioural roles. Sexual dimorphism is another factor implicated in differences between the sexes in terms of their morphology such as, body weight, lung size and capacity. These three variables; biological, social and morphological, can intersect with other factors such as age and ethnicity in influencing the incidence, morbidity and mortality of male/female differences in airway disease. In spite of these sex and gender differences, a recent survey among European physicians confirmed low participation (< 20%) of female individuals in clinical trials and more than 60% reported the lack of sex-specific clinical guidelines [5]. Moreover, drug development and big pharma clinical trials have often excluded women, mainly out of concerns for reproduction and teratogenicity, or even gender bias [6, 7], resulting in adverse drug reactions in women [8]. The situation is changing, however, in clinical and basic research [9], particularly since major funding agencies and scientific journals require sex-differentiated data in studies where sex biology and hormones can play a role in physiological and pathophysiological outcomes [10, 11].

Sex differences in lung diseases may manifest in various biological aspects, including anatomy, physiology, genetics, and hormones [12]. Sexual dimorphism can impact the development of airway diseases through lung capacity and airway size (dysanapsis) [13]. Men tend to have larger airways than women, which can affect airflow and the susceptibility to certain airway diseases. For example, women might be more prone to airway constriction in conditions like asthma due to their relatively smaller airways. Men generally have larger lungs and greater lung capacity compared to women. This anatomical difference can influence the severity and progression of lung diseases like asthma and chronic obstructive pulmonary disease (COPD) [14]. Dysanapsis may be even more important in conditions associated with genetically smaller airways as found in cystic fibrosis. Sex hormones such as estrogen and testosterone can also influence airway diseases [15]. For instance, estrogen might contribute to airway inflammation and bronchoconstriction in women with asthma [16], while testosterone may have protective effects in men [17]. Sex differences have also been observed in cystic fibrosis (CF) and bronchiectasis with females having more severe pulmonary inflammation and more frequent and virulent bacterial infections than CF males [18, 19]. Estrogen has also been shown to exacerbate CF lung disease through effects on ion channels to dehydrate the airway surfaces [20]. Moreover, fluctuations in estrogen levels during the menstrual cycle can impact the severity of airway symptoms in CF [21]. Some women experience worsened respiratory symptoms during the ovulatory phases of their cycle associated with high plasma levels of estrogen. Sex disparities in airway disease also extend to the immune system [22, 23], with differences in immune responses, particularly the immunogenic roles of estrogen and testosterone in asthma among females and males, respectively [24, 25]. This can affect susceptibility to respiratory infections and the development of immune-mediated exacerbations in asthma [26] and CF [27]. Differentially expressed genes between the sexes, in particular, genes coding for inflammation signalling pathways have been reported in asthma and CF [28]. Genetic factors can also contribute to the development of airway diseases from birth, the most well-known being mutations in the gene coding for the cystic fibrosis transmembrane conductance regulator (CFTR) Cl^−^ ion channel in cystic fibrosis which differentially influences CF lung disease morbidity and mortality in males and females [29]. Sex differences are also evident in the rates of pathogen infections [30–32] which play a major role in CF lung disease [33]. Environmental and social factors cannot be neglected, although these are ranked as gender differences in life-style behaviours such as smoking patterns, occupational hazards and social factors which can contribute to variations in airway disease prevalence and severity between sexes, especially, for COPD [34]. We mention this as a cautionary note since gender and sex differences are often confused as interchangeable terms in the literature leading to misinterpretations of the distinct roles of physiological and social influences in disease [35]. That said however, all of the factors underlying biological sex differences and gender diversity in lung pathologies can influence how individuals respond to treatments for airway diseases. Moreover, it is important to note that while these general trends exist, there is significant individual variation, and not all individuals will conform to these patterns. Medications might have different effects or efficacy based on sex-related factors. Different symptom presentations in males and females can lead to diagnostic challenges, potentially resulting in under-diagnosis or misdiagnosis of certain airway diseases. Understanding biological sex differences in airway diseases is crucial in providing personalized and effective medical care [1]. The biological factors behind sex differences in airway diseases are multifactorial and have been recently reviewed separately for CF [18], asthma [36], COPD [37], COVID-19 [38] NCFB (Non-CF bronchiectasis) [39], and LAM (Lymphangioleiomyomatosis) which is almost exclusively confined to women [40]. Here we focus on neglected aspects of sex differences in airway diseases that involve estrogen in the regulation of airway liquid dynamics in CF, asthma and COVID-19, and highlight new avenues of research.

## Steroid sex hormones and sex differences in airway disease

**Fig. 1:**
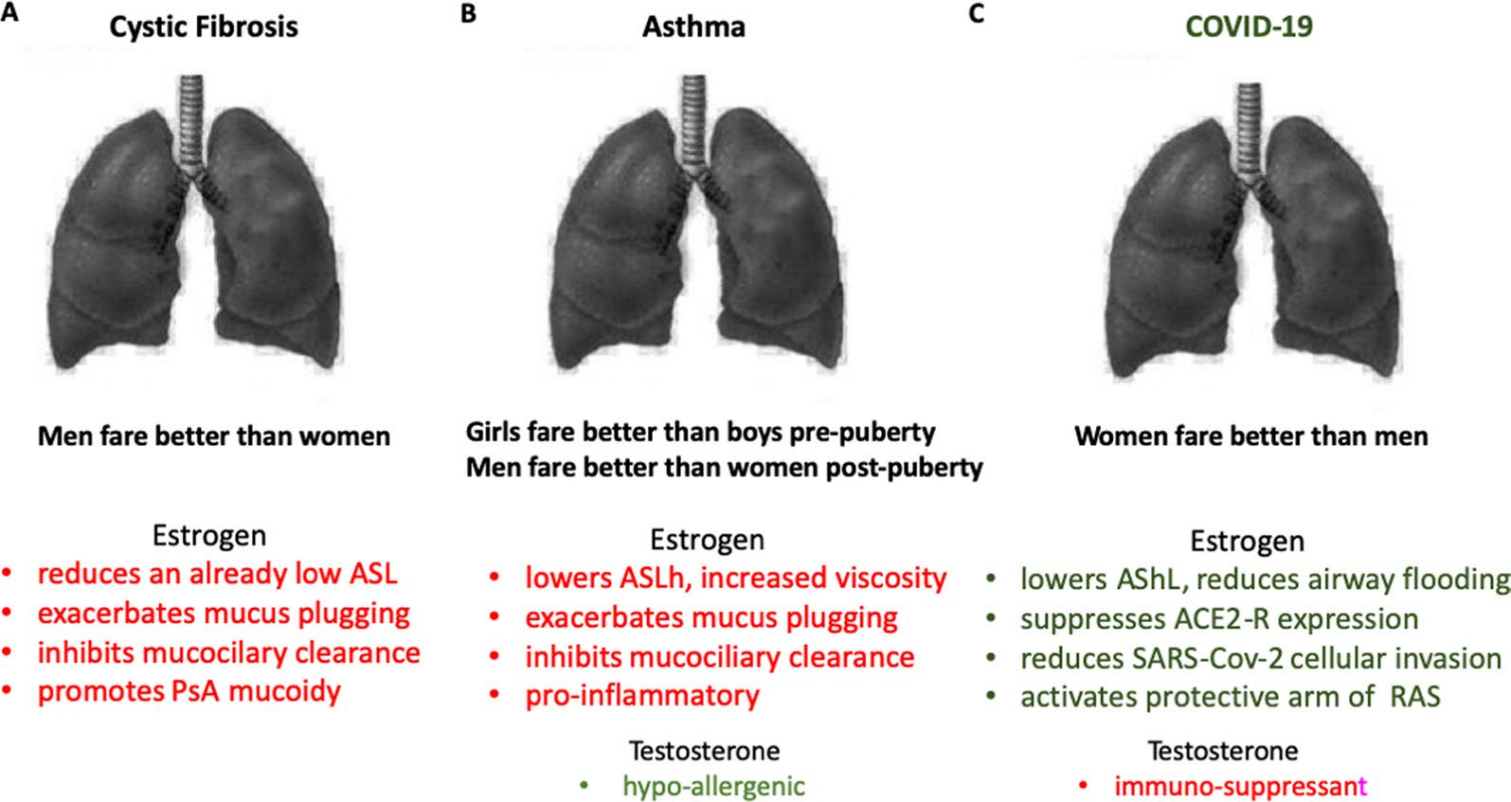
Sex differences in airway disease. **A** In cystic fibrosis, women have worse lung function exacerbations and higher mortality than men. Estrogen has aggravating effects on airway surface liquid dynamics in CF to reduce mucociliary clearance, increase mucus secretion and promote bacterial invasion and virulence. **B** In asthma, boys have worse lung exacerbations than girls pre-puberty, whereas women have more frequent and aggravated asthma episodes than men post-puberty. During pre-puberty, boys have dysanaptic lung development (larger and more hyperactive airways). During post-puberty, estrogen dehydrates the airway surface liquid and promotes mucus plugging of the airways and is hyper-allergenic whereas testosterone is hypo-allergenic. **C** In COVID-19, women have less severe morbidity and mortality than men. Sex steroids may account for this female advantage through several mechanism; X-linked genes offer enhanced immuno-protection and estrogen causes the suppression of ACE2-R expression. Together these responses reduce SARS-Cov-2 cellular invasion, inhibit inflammatory cytokines, activate the protective vasodilatory arm of RAS, and inhibit airway secretions to lower ASL height (ASLh) and reduce airway flooding. Testosterone, on the other hand, increases ACE2-R expression and is immuno-suppressant

Steroid sex hormones play significant roles in the biological sex differences of airway diseases such as cystic fibrosis, asthma, and COVID-19 [18, 26, 38]. Sex differences in these airway diseases are well documented for their biological and clinical divergent responses between males and females in terms of molecular and cellular mechanisms, disease incidence, severity and mortality [41, 42]. The physiological actions of sex hormones are myriad and complex in producing such sex differences in CF, asthma and COVID-19, as summarised in Fig. 1. Biological sex differences in these diseases have been attributed to hormonal (estrogen and testosterone) [2, 13], genetic (XY, XX chromosome genomic imprinting) [43], morphological (dysanapsis) [44], immune (allergen sensitivity), environmental (air pollution) and lifestyle (diet, smoking) factors [24]. The majority of published original research and review articles have focused on the effects of these factors, either together or separately, on airway mechanics (airflow), airway reactivity (bronchospasm), airway infection and inflammation, pharmacotherapy efficacy, and clinical outcomes, while, alas, often neglecting sex differences even when discussing novel therapies [45].

A male advantage is apparent in CF and asthma throughout the reproductive years, whereas a female advantage is seen in asthma pre-puberty (Fig. 1A, 1B), [46, 47]. A female advantage has also been observed in COVID-19 (Fig. 1C), although the incidence is similar between the sexes [37]. The variability in the severity of these diseases among the sexes during pre- and post-puberty and pre-and post-menopause point to a major role of the female and male sex steroid hormones, estrogen and testosterone, respectively, in the aetiology of asthma, CF and COVID-19 lung disease. Female sex hormones, in particular estrogen (E2, 17β-estradiol) have been implicated in modulating the onset, development and progression of airway disease in CF [48], asthma [26], COPD [2] and COVID-19 [49, 50]. Estrogen regulates various factors underlying sex differences in these airway diseases through modulating immune responsiveness, microbial infections, and airway surface liquid dynamics.

There has been recent debate whether sex differences in airway diseases has subsided as a result of improved healthcare, lifestyle and targeted therapies [51]. The majority of studies point to a persistent trend in sex differences in CF and asthma, and in bronchiectasis in general [1], underpinned by actions of sex steroids in the airways. Cystic fibrosis is probably the most well-studied airway disease in terms of sex differences in which the question of whether or not biological sex is a risk factor. Some of the first early studies in the 1990s reported decreased survival rates among adult female CF patients [52] and poorer lung function associated with higher rates of mucoid *Pseudomonas aeruginosa* [33]. These findings were confirmed in large multicentre studies which found young (< 20y) female CF patients were 60% more likely to die than age-matched CF males [53]. The so called CF (and misnomer) ‘Gender Gap’ has persisted into the twenty-first century with several studies showing worse quality of life, pulmonary exacerbations and mortality outcomes for CF females than males with CF [54]. The most recent data from the Canadian CF Registry 2022 has confirmed this continued trend [55] and the latest clinical data from combination CFTR modulator therapy, elexacaftor/tezacaftor/ivacaftor still show worse pulmonary exacerbations in female CF patients compared to males, despite greatly improved lung function in both sexes [56]. These findings concur with data from non-CF bronchiectasis (NCFB), which, in most countries, is female-predominant and where females are more symptomatic, and have lower lung function and present earlier [19, 57].

## Estrogen in airway diseases

**Fig. 2:**
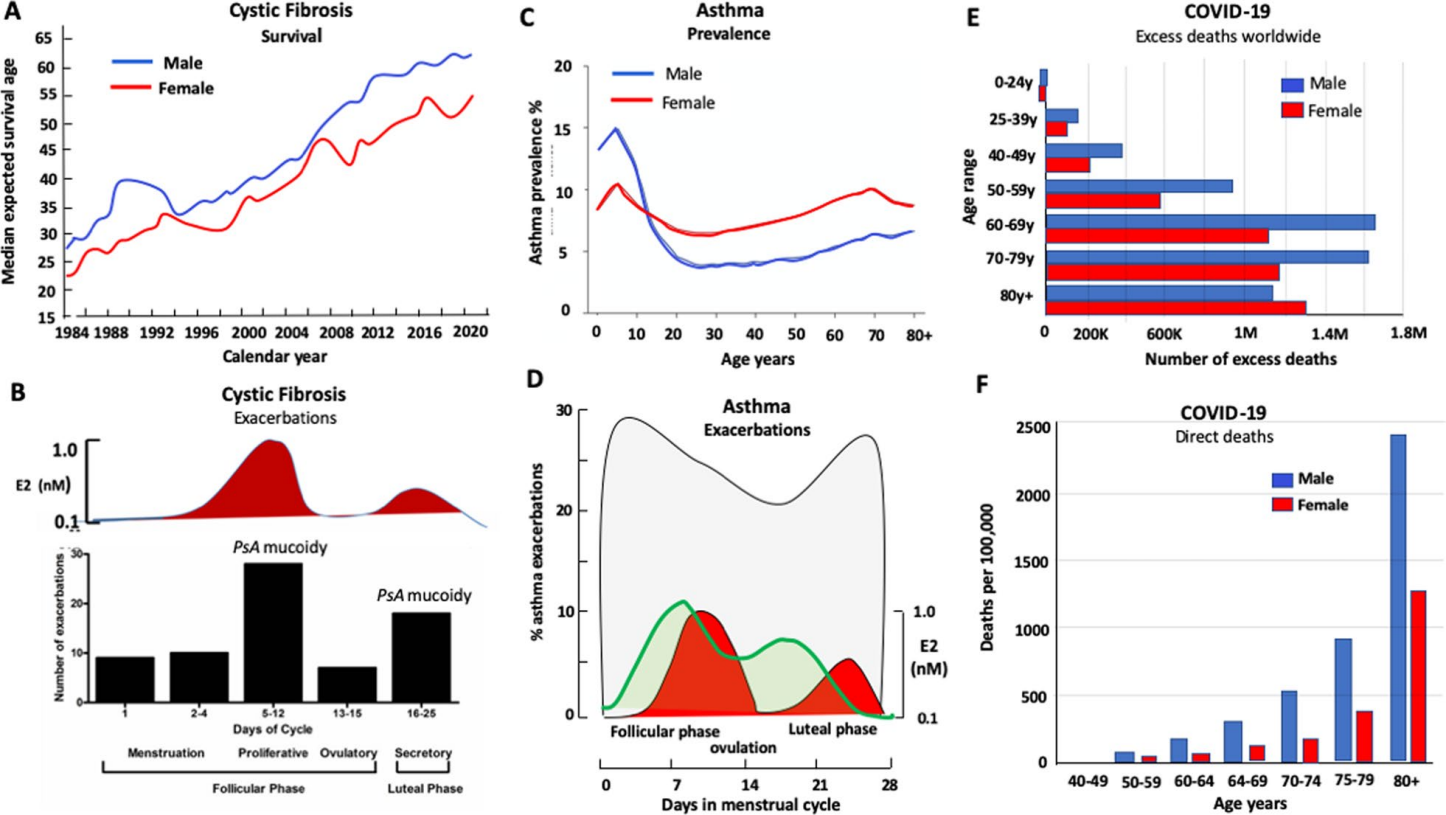
Mortality and pulmonary exacerbations in airway diseases are sex-dependent. **A** Cystic fibrosis: Estimated median age of survival for a moving 5-year window with 95% confidence intervals, by sex, 1984 to 2020 in Canada. The sex difference in CF survival and mortality outcomes has been found in many other countries including Australia, France, UK and USA. Adapted and redrawn using data from Cystic Fibrosis Canada. (2022). *The Canadian Cystic Fibrosis Registry 2020 Annual Data Report*. Page 44, Retrieved on 10 October 2023 from https://www.cysticfibrosis.ca/Registry/2020AnnualDataReport.pdf. **B** Changes in estrogen and lung exacerbations are correlated throughout the menstrual cycle. Lung exacerbations (determined from Forced expiratory volume in 1 s (FEV_1_) and forced vital capacity (FVC)) increase during peak plasma levels of 17β-estradiol (E2). During the menstruation phase, estrogen levels are low and pulmonary exacerbations are reduced. In the proliferative phase, estrogen begins to rise and peak just prior to ovulation when rates of pulmonary exacerbations are highest which then fall during ovulation and increase again during the luteal phase. Other factors contributing to lung exacerbations during high estrogen states include a more mucoid *P. aeruginosa* phenotype, elevated markers of inflammation (tumour necrosis factor-α (TNF-α), interleukin-8 (IL-8) and free neutrophil elastase (NE). **C** Asthma prevalence percentage throughout life in developed countries. Graph based on the most up-to-date data available (2018) from the Global Health Data Exchange (https://ghdx.healthdata.org). **D** Asthma exacerbations (grey shaded area) according to phase of menstrual cycle and plasma E2 levels (red shaded area) when classified by symptom onset or day of emergency department visit. Asthma exacerbations began more often during the preovulatory (28%) and peri menstrual phases (27%) of the cycle than in the periovulatory or postovulatory phases. Hyper-immunoreactivity in follicular and luteal phases is shown in green shaded area corresponding to measured high TNFα, mast cells, hyper-eosinophilia and free neutrophil elastase. **E** Mean excess deaths associated with the Covid-19 pandemic worldwide classified by sex (female red stacks, male blue stacks) and age range. Data accessed 3 October 2023 from: https://www.statista.com/statistics/1306958/number-excess-deaths-covid-pandemic-by-age-and-gender-worldwide/. **F** Deaths attributable directly to COVID-19 grouped by age and sex from 8 representative countries (female red stacks, male blue stacks). The data from different countries show that male mortality is higher than females. The data for Peru, Italy, Spain, England, France, USA, and Mexico were obtained from Global Health 50/50 which was updated July 12 2023. The mortality data as of July 24 2023 for Sweden was taken from the Swedish Public Health Agency. Adapted and redrawn using data from: The COVID-19 Sex-Disaggregated Data Tracker. https://globalhealth5050.org/the-sex-gender-and-covid-19-project/the-data-tracker/?explore=variable&variable=Deaths. The Disaggregated Data Tracker is updated every month, the graph shows the last update when accessed on 3 October 2023

Several studies have provided evidence for an endocrine basis for sex differences in airway diseases where a marked advantage for one of the sexes over the other is apparent from puberty (Fig. 2). The biological mechanisms behind these sex differences are multifactorial and involve hormonal, genetic, and immunological factors. Hormones, such as estrogen, progesterone and testosterone, have been shown to influence airway inflammation and immune responses. Additionally, genetic and epigenetic factors may contribute to sex-specific vulnerabilities in the development and progression of airway diseases.

Sex differences still prevail in the prevalence, severity, and clinical presentation of CF, asthma and COVID-19 airway diseases. As stated above, sex differences have been shown to persist in cystic fibrosis since sex-disaggregated data were first documented in the 1970s. Female CF patients have worse morbidity than age-matched males and present with earlier and more severe colonisation of the airways by pathogens *Pseudomonas aeruginosa* and *Bukholderua cepia* pathogens, and more severe and frequent lung exacerbations and shorter life expectancy than age-matched males (Fig. 2A), [58]. The fact that these deteriorations in lung physiology occur around the age of puberty point to a role of sex steroid hormones, notably estrogen in females. Moreover, the frequency and severity of lung exacerbations in female CF patients fluctuate within the estrous cycle with most severe events occurring during the ovulation phase of proestrus and estrus when plasma levels of estrogen are highest (Fig. 2B), [21]. Pregnancy, although rare in CF females, is associated with more severe and frequent occurrence of lung exacerbations [59]. In contrast, female CF patients taking the combined oral contraceptive pill showed a reduction in lung exacerbations down to a level observed in males [21, 58]. These observations taken together, point to a pivotal role for estrogen in causing depressed respiratory lung function in female CF patients compared to males.

In childhood asthma, boys show more severe allergic asthma reactions and frequent episodes than young girls pre-puberty. These sex differences are reversed around the age of puberty with worse asthma symptoms observed in females up to the menopausal years (Fig. 2C). Asthma exacerbations have been found to be co-incident with fluctuating high plasma estradiol levels [60, 61]. Severity and frequency of asthma episodes show dependency on the stage of the menstrual cycle, with worse lung function during proestrus and estrus phases, which are associated with hyper-immunogenic states (Fig. 2D), [62]. Moreover, post-menopause hormone replacement therapy (HRT) is reported to alleviate asthma in females, whereas pregnancy is associated with more severe asthma episodes [63, 64], all pointing to a pivotal role for estrogen.

Clear sex differences with a female advantage in COVID-19 mortality have been documented in every WHO country since the beginning of the pandemic [65]. The beneficial effects of female sex in COVID-19 have been documented since the outbreak of the pandemic in every country where sex disaggregated data are available [66]. The excess death rate attributable directly to COVID-19 has been less frequent in age-matched females (Fig. 2E), and the lower mortality is not apparent until post-puberty during the reproductive years (Fig. 2F). These female sex-specific responses across a wide spectrum of airway diseases point to a key role of the female sex steroid hormone estrogen or to be more precise, its biological active form 17β-oestradiol (E2), underpinning sex differences in airway diseases.

## Estrogen regulated ion channels in airway disease

Estrogen, a hormone primarily associated with female physiology, can influence various physiological processes related to the airways and respiratory system. Estrogen receptors are present in the airway epithelial cells, smooth muscle cells, and immune cells, along with estrogen-sensitive ion channels, suggesting that estrogen can impact the function of ion channels within the airways [67, 68]. Dysregulation of estrogen modulated ion channels has been associated with various airway diseases [69] and are discussed in detail in this section.

### Estrogen regulated ion channels in CF

Cystic Fibrosis is an inherited genetic disorder caused by mutations in the CFTR gene, which encodes a chloride ion channel [70]. CFTR is essential for proper hydration of the airways and is key for generating an efficient mucociliary clearance [71]. CF is characterized by the production of thick, sticky mucus that obstructs the airways and leads to chronic respiratory infections, bronchiectasis and progressive lung damage [72]. The disease primarily affects the lungs, and despite significant advances in treatment, CF remains a life-limiting condition (current median life expectancy is 50 years). In recent years, there has been increasing interest in understanding the role of estrogen in CF airway disease, as estrogen actions in exacerbating various physiological processes, including airway hydration and clearance, immunity and inflammation, have implications for CF disease progression and therapeutic interventions. While estrogen’s direct influence on CFTR is not well understood, hormonal changes during puberty and the menstrual cycle have been associated with changes in CF symptoms. CF exacerbations in the female have been associated with high estrogen states and studies have shown estrogen enhances mucus secretion [73], and triggers hyper-inflammatory responses to bacterial infections and promotes a mucoid *Pseudomonas aeruginosa* (PsA) phenotype [21], higher infection rates of PsA [74] and *Staphylococcus aureus* [75], and worsens bacterial virulence [76]. The hallmark of CF lung disease is the overproduction of thick mucus, leading to impaired mucociliary clearance. Estrogen has been found to influence mucus production and clearance mechanisms in the respiratory epithelium by modulating the expression and activity of ion channels and transporters responsible for maintaining airway surface liquid hydration, which directly affects the consistency (viscosity) of mucus [73]. Moreover, estrogen actions to reduce airway surface liquid height (ASLh) [77, 78] and inhibit ciliary beat frequency [76] may further impede mucociliary clearance, potentially increasing the risk of chronic bacterial colonization and infections in CF patients.

### Estrogen regulated ion channels in asthma

Asthma is a chronic respiratory disease characterized by airway inflammation, bronchoconstriction, hyper-responsiveness and increased mucus production, leading to recurrent episodes of wheezing, breathlessness, and coughing. While asthma is more prevalent in females after puberty, the role of estrogen is still not well understood, although the influence of estrogen regulated ion channels is becoming more apparent in underpinning sex differences in asthma. In this context, ion channels play a critical role in airway smooth muscle contraction, mucus production and cell signalling which can contribute to airway hyper-responsiveness and inflammation in asthma [79]. For instance, certain ion channels, such as calcium channels, are involved in controlling the contraction of airway smooth muscle. Abnormalities in these ion channels can lead to excessive muscle contraction and bronchoconstriction, hallmarks of asthma [80]. Estrogen has been shown to influence the expression and activity of ion channels involved in bronchial smooth muscle contraction, immune responses, and mucus production [36, 68, 69, 81–83]. For example, estrogen can modulate calcium-activated chloride channels and potassium channels in bronchial smooth muscle cells, influencing intracellular calcium levels and cell excitability to enhance airway tone [84]. Estrogen effects on immune responses can influence susceptibility to respiratory infections and allergic reactions. Estrogen exerts a complex influence on the immune system, impacting both innate and adaptive immune responses. Studies have revealed that estrogen can enhance the production of type 2 cytokines, such as interleukin-4 (IL-4) and interleukin-5 (IL-5), which play critical roles in promoting airway inflammation and allergic responses associated with asthma [67]. Estrogen-regulated ion channels in immune cells can influence airway hyper-immune and inflammation reactions. Estrogen has been shown to modulate voltage-gated sodium and potassium channels and Transient Receptor Potential (TRP) channels in immune cells, which play a crucial role in bronchial hyper-responsiveness and could potentially impact the immune response to infections and allergens, and contribute to the severity or frequency of asthma episodes [85]. The modulation of ion channel activity by estrogen could potentially contribute to the sex dependency observed in some aspects of asthma airway disease.

### Estrogen regulated ion channels in COVID-19

COVID-19, caused by the novel coronavirus SARS-CoV-2, primarily affects the respiratory system, leading to symptoms that range from mild upper respiratory symptoms to acute respiratory distress syndrome (ARDS). The COVID-19 pandemic has spurred extensive research into the mechanisms of SARS-CoV-2 infection, disease progression, and potential treatments. Among the factors under investigation, the “gender gap” or female advantage, and the role of sex hormones, particularly estrogen, in COVID-19 have garnered significant attention [86–88]. In this context, we explore the potential impact of estrogen on ion channels influencing COVID-19, while acknowledging the complex and multifaceted nature of this disease. The SARS-CoV-2 virus enters human airway cells through the interaction between its spike protein and the angiotensin-converting enzyme 2 receptor (ACE2-R) on the surface of host cells. This interaction can lead to changes in ion channel activity, specifically calcium channels, which play a role in regulating intracellular signalling pathways involved in cytokine production and immune cell activation [89]. Changes in calcium ion channel activity may contribute to the immune response and inflammation seen in COVID-19. Estrogen has been shown to downregulate ACE2-R expression on the cell membrane surface and thus provide a mechanism to reduce viral entry into airway epithelia. ACE2-R also regulates estrogen receptors thus reinforcing sex dependency in COVID-19 [90]. The potential protective effects of estrogen in lung and cardiovascular pathologies associated with COVID-19 are not limited to ACE2-R expression and may also involve transmembrane protease/serine protease 2 [TMPRSS2], ADAM metallopeptidase domain ADAM-17 and ACE2 expression [91]. TMPRSS2 activates the SARS-CoV-2 spike protein while ADAM-17 decreases the levels of membrane-bound ACE2 and in this way inhibits the process of SARS-CoV-2 cell entry. The effects of estrogen to favourably modulate the expression of these factors to limit viral entry may contribute to female protection against acquiring severe COVID-19 disease [92]. COVID-19 has been associated with a higher risk of blood clotting and thrombosis, exacerbated in part by overstimulation of the renin angiotensin system (RAS) vasoconstriction pathways. Estrogen has been shown to inactivate the vasoconstrictor arm of RAS and stimulate vasorelaxation [93]. The mechanisms of action involves estrogen inhibition of ACE through upregulation of renin and angiotensinogen thereby increasing angiotensin I (AngI- vasodilation) and suppressing angiotensin II (AngII-vasoconstriction). Thus hypertension can be prevented in high estrogen states with alternative processing of angiotensinogen to Ang I rather than to Ang II. Vasodilation can also be enhanced in females by estrogen activation of eNOS and estrogen-regulated K^+^ channels in endothelial cells and vascular smooth muscle [94]. There is a caveat here arising from a recent study that reported an increased venous thromboembolism (VTE) in COVID-19 patients undergoing treatment with estrogen containing oral contraceptive pill (OCP) [95]. However, this risk was highest for patients over the age of 50 (potentially post-menopause) and was not significantly associated with oral estrogen. It is quite likely the increased VTE observed in these patients may have resulted from OCP feedback inhibition of luteinizing hormone and concomitant reduction in free plasma oestradiol levels which are normally cardio-protective. Moreover, a similar recent survey has noted no significant VTE risk with the use of OCP in women with COVID-19 [96].

Severe cases of COVID-19 are often characterized by a cytokine storm, an excessive and dysregulated immune response. Estrogen-regulated ion channels can play a role in the activation and regulation of immune cells, including estrogen inhibition of inflammatory transcription factors (NFkB) and inflammatory cytokines (IL-8) [27], and estrogen activation of anti-inflammatory cytokines (IL-4, IL-10) and helper T-cells and B-cells [97]. Pulmonary edema is still one of the main causes of mortality in Covid-19 and the severe form of the disease is associated with lung hypersecretion and extravascular fluid accumulation [98]. Indeed, there was concern at the very start of the pandemic for flash-pulmonary edema, so fluid resuscitation was kept to a minimum. Increased extravascular lung water (EVLW), an index of flooding of the airways, reflects long ICU stays and mortality in COVID-19 associated ARDS [99]. It is likely that failure in alveolar and bronchiolar fluid clearance plays a major role in the pathogenesis of COVID-19 pulmonary edema. The imbalance of airway hypersecretion, increased pulmonary vascular permeability and massive fluid exudation driven by Starling forces into the lung, combined with low pulmonary fluid clearance and increased colloidal pressure (a protein-rich EVLW), may be key reasons for the acute exacerbation of pulmonary edema and increased ASL volume in COVID-19 patients**.** While direct research on airway surface liquid volume in COVID-19 is limited, there are several reasons why it could be of interest in explaining sex differences in COVID-19 morbidity and mortality: Efficient mucus clearance is crucial for the prevention of respiratory infections. Alterations in ASL height could impact the ability of cilia to clear mucus, potentially affecting the lung's capacity to clear viral particles, including SARS-CoV-2. A well-hydrated ASL is considered to be more effective at trapping and removing pathogens. Changes in ASL height may influence susceptibility to respiratory infections in COVID-19. Severe cases of COVID-19 are associated with an exaggerated immune response and cytokine storm and ASL height and the state of the airway epithelium may influence the local immune response in the respiratory tract. While ASL height regulation is a well-studied aspect of respiratory physiology, its specific role in the pathogenesis of COVID-19 remains unclear. Future research may shed light on whether alterations in ASL height contribute to the susceptibility, severity, or outcomes of COVID-19, and whether targeting ASL volume regulation could be a potential therapeutic approach for respiratory viral infections. In the following sections we will explore the regulation of ASL dynamics by estrogen and compare with the actions of corticosteroids, in particular, dexamethasone which is used effectively to reduce ICU admissions and mortality in severe COVID-19 [100].

## Airway surface liquid and mucociliary clearance

The airway surface liquid (ASL) is a thin hydrated layer bathing the airway epithelial cell surfaces and is composed of the periciliary layer (PCL) and a mucus layer overlying the PCL. The ASL is a critical component of the respiratory system and serves several vital functions: It acts as a protective barrier, trapping and removing inhaled pathogens, particulate matter, and foreign substances. A well-hydrated ASL facilitates the movement of cilia, tiny hair-like structures on the airway epithelial cells, which help clear mucus and debris from the airways. In the healthy lung, the ASL contains anti-bacterial compounds to fight infection and sustains the proper hydration of the airway epithelium, ensuring efficient gas exchange. Under physiological conditions, the ASL is maintained at an optimal height just covering the length of outstretched cilia (a periciliary liquid layer of 7–10 µm equivalent to 1 µL/cm^2^ of mucosal surface) [101]. The ASL height (ASLh) is generated by transepithelial ion transport with passive water flux following the osmotic gradient. The molecular mechanisms which sense the optimal ASLh are still unknown, whether they be osmotic stimuli, cilia tension, ion channel regulators or hormonal/paracrine signalling [102–109].

**Fig. 3:**
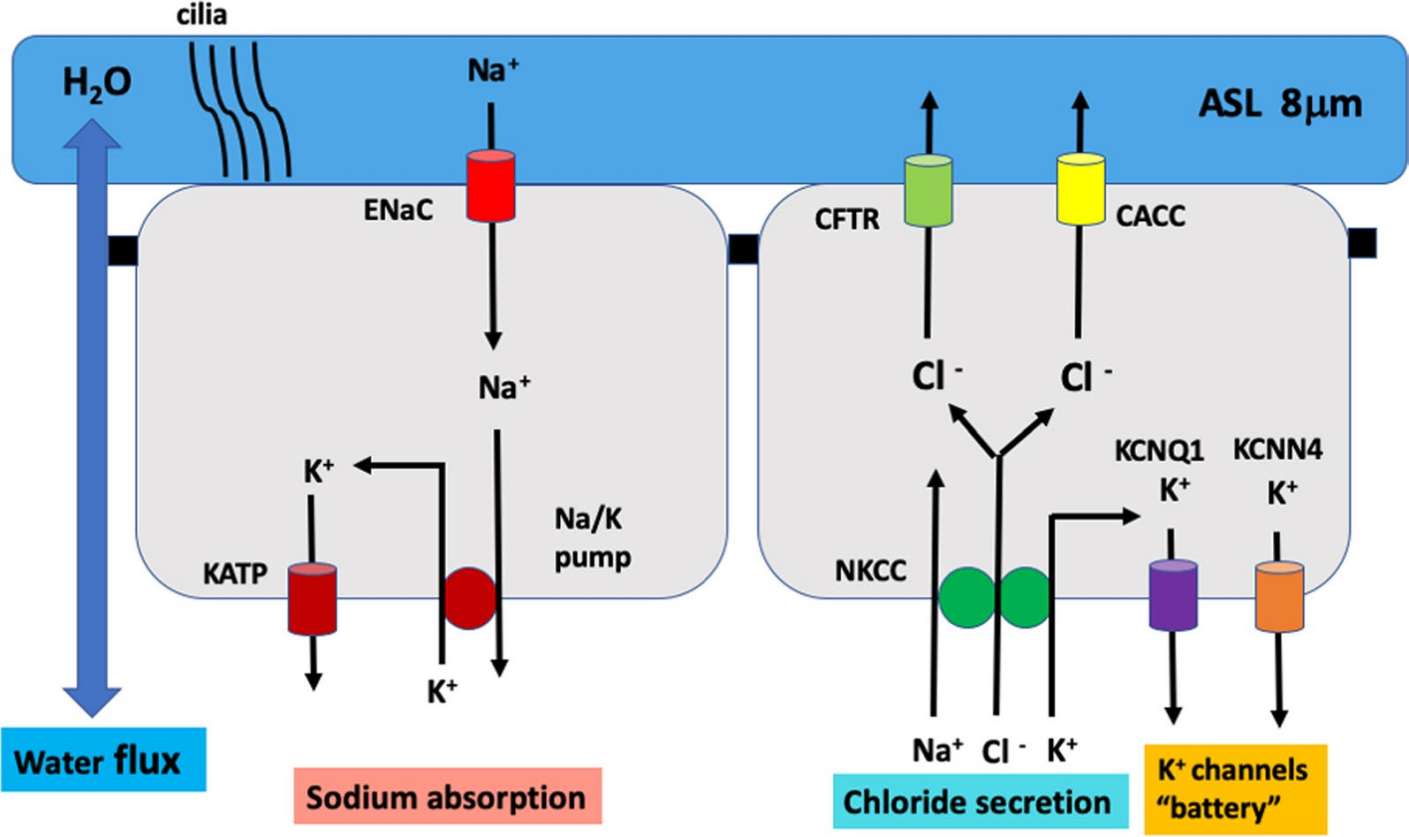
Ion transport generates the airway surface liquid height. Sodium ion absorption and chloride ion secretion generate an osmotic gradient for water flux across the airway epithelium. In healthy airways, the transepithelial transport of Na^+^ and Cl^−^ is balanced so as to generate an optimal ASLh between 7 and 10 µm corresponding to the length of outstretched cilia. Sodium absorption occurs as a two-step process with sodium entry via ENaC Na^+^ channels in the apical membranes and then Na^+^ is pumped out of the cells into the blood side via Na/K-ATPase in the basolateral membranes. The electrical charge balance for Na^+^ absorption is generated by the activity of KATP (Kir6.1) potassium ion channels in the basolateral membranes. Inhibition of any one of these transporters (ENaC, Na/K-ATPase, KATP) will tend to decrease sodium and water absorption, and as a consequence increase ASLh, whereas stimulation of the Na^+^ transport pathways will decrease ASLh. Chloride ion secretion also occurs as a two-step process, with Cl^−^ entering the airway epithelial cell across the basolateral membranes via the Na:K:2Cl cotransporter and then transported down an electrochemical gradient into the ASL via CFTR Cl^−^ channels which support basal and cAMP-stimulated Cl^−^ secretion. Calcium-activated CaCC Cl^−^ channels are the pathway for calcium-activated Cl^−^ secretion. The charge balance for CFTR-mediated Cl^−^ secretion is provided by the cAMP activated KCNQ1:KCNE3 K^+^ channel, and for CaCC channels via calcium-activated KCNN4 K^+^ channels. Inhibition of any one the chloride ion secretion pathways will decrease water flux into the ASL and reduce ASLh, whereas stimulation of chloride ion secretion transporters will increase ASLh

The ion transporter mechanisms generating the ASL are well-described and are summarised in schematic form in Fig. 3. The optimal ASLh is the result of a balance between net Cl^−^ secretion and net Na^+^ absorption, with water flux following an osmotic gradient. Transepithelial Cl^−^ secretion occurs by a two-step process with Cl^−^ entry into the airway epithelial cells from the blood side across the basolateral cell membranes via a Na:K:2Cl cotransporter (NKCC). Chloride ions then exit the cell across the apical membranes via cAMP-sensitive CFTR and calcium-activated Cl^−^ channels (CaCC) [110]. The charge balance for Cl^−^ exit via CFTR is provided by cAMP-sensitive K^+^ channels (KCNQ1:KCNE3) whereas Cl^−^ flux via CaCC is balanced by calcium-dependent K^+^ channels (KCNN4) [111–114].

Transepithelial Na^+^ absorption is also a two-step process conforming to the Ussing model. Sodium ions enter the cell from the airway side via the epithelial Na^+^ channel (ENaC) and exit the cell across the basolateral membranes via the Na/K-ATPase pump. Charge balance for sodium ion absorption is dependent on recycling of K^+^ via inwardly rectifying ATP-sensitive K^+^ channels (Kir6.1, KATP) [112, 115]. The activity of the Na/K-ATPase pumps is vital in maintaining favourable electrochemical driving forces for all sodium and chloride ion transport routes. Transepithelial water flux mainly takes a cellular route, via aquaporin water channels located in apical and basolateral membranes. Net water flux can go in either direction, into or out of the airways, depending on the osmotic gradient generated by Na^+^ absorption and Cl^−^ secretion [116]. Activation or inhibition of any one of the ion channels, transporters or pumps along the transcellular routes will affect the absorption and secretion rates for Na^+^, Cl^−^ and water, and by consequence will modulate the ASL height and volume.

The generation of the optimal ASLh and ciliary beating is essential for effective mucociliary clearance. Mucociliary clearance is a key physiological component of airway health to clear the large and smaller airways of invasive pathogens, excess mucus and inspired inflammatory particles [117]. This mucociliary ‘escalator’ is dependent on effective airway surface liquid dynamics involving the generation of an optimal ASLh, and the efficient beating of cilia [118–120]. During an active stroke, the cilia make contact with the overlying mucus layer and propel mucus and trapped pathogens towards the mouth at a rate of ∼3 mm.min^−1^ [121]. In this way, the airways are kept relatively sterile and clean of irritants. The generation of ASLh and ciliary beating are under the control of ion channels which regulate, respectively, the electrical driving force for electrolyte and water transport across the airway epithelium, and calcium and kinase signalling essential for the ‘whiplash’ stroke of cilia [113, 122, 123]. Dysfunction in the regulatory mechanisms of mucociliary clearance can contribute significantly to airway diseases, including cystic fibrosis, COPD, asthma and COVID-19.

### ASL dynamics in airway disease

Here we define ASL dynamics as the components of the layer of fluid overlying the airway surfaces which regulate mucociliary clearance: the ASL height and viscosity, ciliary beat frequency, mucus secretion and pathogen load, and other intrinsic factors such as ASL pH, channel activating proteases, defensins, inflammatory cytokines, specialised pro-resolution lipid mediators and glucose concentration. A dysregulation of ASL dynamics is a common feature of CF [124–128], asthma [129–131] and COVID-19 [132–135]. In the following sections we provide a background to ASL dynamics in airway diseases and review the evidence for estrogen regulation of the component properties of ASL dynamics and how these estrogenic responses may contribute to biological sex differences in these diseases.

### ASL dynamics in cystic fibrosis

**Fig. 4:**
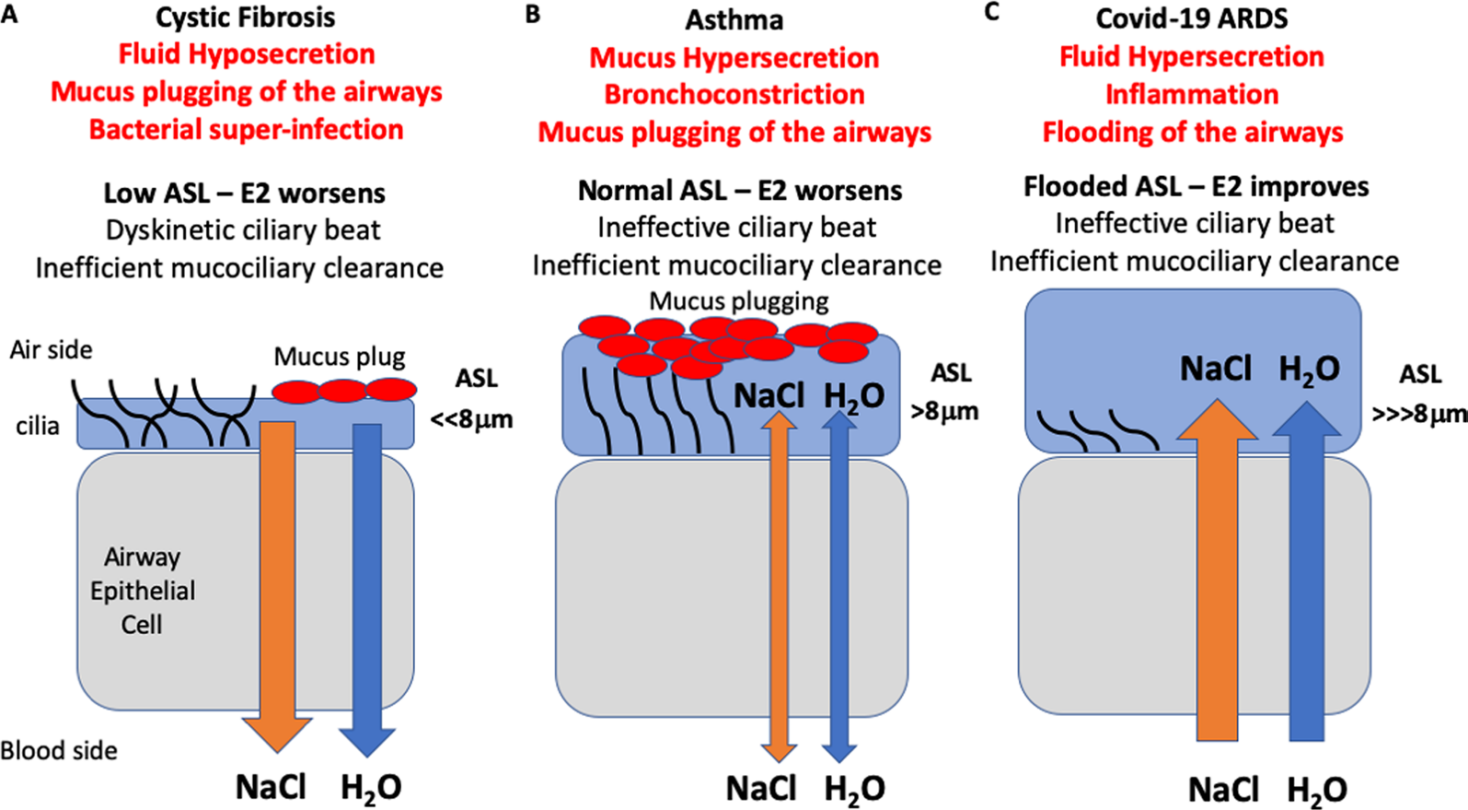
ASL dynamics in airway diseases. **A** Cystic fibrosis is characterised by extremely low rates of Cl^−^ secretion and a hyperabsorption of Na^+^ which can dehydrate the airway surface fluid and produce a low ASLh, dyskinetic ciliary beat, mucus plugging, inefficient mucociliary clearance and bacterial virulence. Estrogen (E2) exacerbates each one of these parameters and worsens CF lung disease in female patients. **B** The hallmarks of asthma are mucus hypersecretion, inflammation, and bronchoconstriction. ASLh may be normal in the asthmatic lung but mucociliary clearance is hampered by a viscous ASL and mucus plugging. Estrogen can aggravate this condition by inhibiting CFTR Cl^−^ secretion and stimulating ENaC Na^+^ absorption, drawing water out of the airway fluid to further increase its viscosity as well as by stimulating mucus production and secretion. **C** In COVID-19, the SARS-CoV-2 virus causes severe lung inflammatory responses and fluid exudation which result in flooding of the alveoli and central airways. The hugely increased ASLh renders mucociliary clearance difficult leading to increased viral load and a vicious cycle of inflammation and airway flooding. Estrogen can alleviate ASL hydration, especially in the alveoli by stimulating Na^+^ absorption (which is inhibited by cytokines and TNFα in the inflammed airways), and contribute to better survival in female COVID-19 patients

Airway surface liquid dynamics is very much compromised in CF, showing increased pathogen virulence, high viscosity, dehydration and disturbances in innate mucosal defence, acid/base balance, glucose metabolism and mucin secretion [127]. CF is characterised by a low ASLh of less than 8 µm. Since the ASLh drops below the height of outstretched cilia, ciliary beat frequency is compromised in CF, resulting in poor mucociliary clearance. Mucus becomes trapped in the airways and clogging is further aggravated by hypersecretion of mucus from submucosal glands. The resultant mucus plugging favours chronic bacterial infection, inflammation and lung damage. There is evidence that CF ASL is acidic and a low pH may compromise bactericidal activity [136]. The low ASLh is caused primarily by a greatly reduced Cl^−^ secretion due to the loss of functional CFTR (mainly a mutation in deltaF508 CFTR) which decreases fluid secretion into the periciliary layer. Airway dehydration is further exacerbated by overactive ENaC channels which increase sodium absorption [137], dragging water out of the periciliary layer [138, 139] (Fig. 4A).

### ASL dynamics in asthma

Asthma is characterised by hypersecretion of mucus as a result of hyperplasia of goblet cells and over-stimulated secretion by submucosal glands [140] coupled with reflex secretion brought on with chronic cough [141]. Unlike CF, airway mucous secretions in asthmatics are comparatively sterile, thus, asthma airway surface fluid may be characterized by large volumes of comparatively normal mucous secretions. Mucociliary clearance is low in hospitalised asthmatics and much reduced in chronic asthmatics, especially from the central airways (where ciliated cells are present) [142]. Pharmacological antagonists of mucin synthesis and exocytosis have variable efficacy in reducing mucus hypersecretion and potential therapies to hydrate airway secretions and increase ASLh are currently being investigated [143–145]. There is, however, little research available on the role played by ASL dynamics in asthma. Most studies have been confined to the role of innate immunity of the airway epithelium in allergic asthma [146], and the only available study showing a reduction of hypertonicity in ASL did not appear to influence thermally induced asthma [129]. Given the known effects of mucus plugging on airway infection and inflammation, we can hypothesise that hormones or drugs which increase ASLh should have a positive effect to enhance mucociliary clearance in asthma, whereas antagonists of ASL secretion should worsen the disease [147] (Fig. 4B).

### ASL dynamics in COVID-19

Severe COVID-19 is characterised by hypersecretion of airway surface fluid and alveolar flooding. The SARS-2-CoV virus is thought to destabilise adherens junctions and disrupt tight junctions in the airway epithelium [148–150]. A leaky epithelium allows plasma transudation which can be exacerbated when combined with increased subepithelial hydrostatic pressure and intraluminal colloid pressure as a result of widespread pulmonary inflammation and cytokine damage to the respiratory epithelium. These factors all contribute to bulk flow of fluid into the lungs in COVID-19 lung disease. Thus hypersecretion and plasma transudation become important contributors to the increased volume of airway surface liquid and flooding of the airways in severe COVID-19 [151] (Fig. 4C).

## Steroid regulation of ASL dynamics

Various steroids have been reported to modulate ASL height and/or ciliary beat. These include sex hormones, corticosteroids including synthetic glucocorticoids, and bile acids. Studies over the past decade have shown that steroid regulation can indeed impact the dynamics of the airway surface liquid in the respiratory system. By far estrogen is the most potent steroid hormone reported to modulate ASLh and ciliary beat and is reviewed in detail in section "Estrogen regulation of ASL dynamics". There are no reports of actions of androgens or progesterone on ASL fluid secretion, however, progesterone has been reported to inhibit ciliary beat frequency in airway epithelium [152]. Bile acids are also implicated in airway disease [153], particularly in CF [154], by activating CFTR and inhibiting ENaC channels to raise ASL height [155, 156].

Glucocorticoid medications are commonly prescribed in the management of respiratory conditions such as asthma, COPD, and more recently in the management of severe COVID-19 [100]. These treatments can have a positive impact on the regulation of the airway surface liquid, helping to maintain proper airway function. In addition to its anti-inflammatory role in airway epithelium, dexamethasone has been shown to modulate the activity of ion channels which generate the ASL, notably to activate ENaC channel subunit insertion into the apical membranes of airway epithelial cells and thus stimulate sodium absorption [157–159]. Dexamethasone has also been reported to modulate chloride secretion [160] across human bronchial epithelium by inhibiting K^+^ channels required for generating the electrical driving force for Cl^−^ exit across apical membranes. The anti-secretory effects of dexamethasone involve a protein kinase A (PKA) dependent reduction in the activity of KCa3.1 (KCNN4) channels which drive calcium-dependent chloride secretion pathways, and a protein kinase C delta (PKCδ) mediated inhibition of KV7.1 (KCNQ1:KCNE3) channels which are essential for CFTR-dependent Cl^−^ secretion [161]. All of these K^+^ channels are highly expressed in human bronchial epithelia [112]. The combined pro-absorptive and anti-secretory effects of dexamethasone can result in water being drawn out of the airways. In fact, this is a physiological response to glucocorticoids at birth. In the fetal lung, the alveolar spaces are filled with fluid to allow gas exchange with maternal blood. Fetal alveolar flooding is dependent upon hyper-secretion of Cl^−^ and hypo-absorption of Na^+^ to produce a net flux of water into the alveolar spaces [162, 163]. Just before birth, these ion transport processes are reversed and fluid absorption is greatly increased due to a stimulation of sodium absorption via activation of ENaC and Na/K-ATPase pumps triggered by a surge in catecholamines and corticosteroids [164, 165]. The net effect is to reabsorb fluid and prevent alveolar edema [138, 166], while producing a thin layered ASL favourable for air breathing. An analogous effect has been proposed for the beneficial effects of dexamethasone to reduce pulmonary edema in severe COVID-19 [167].

## Estrogen regulation of ASL dynamics

**Fig. 5:**
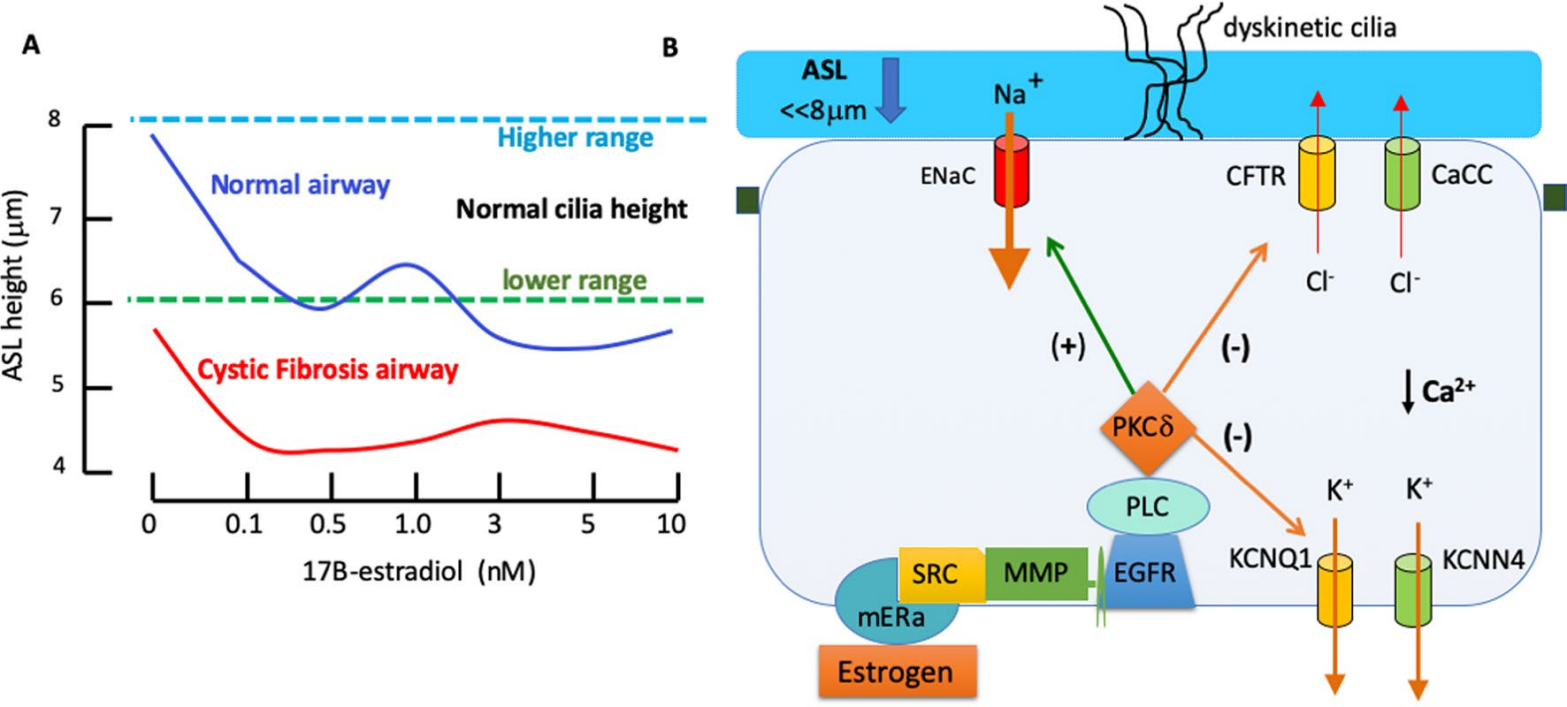
Estrogen modulation of ASL height and cell signalling mechanisms. **A** Changes in ASL height produced by varying concentrations of estrogen (E2, 17β-oestradiol) in human female-derived bronchial epithelia. Low concentrations of E2 (in the range observed during the menstrual cycle) cause a rapid and sustained decrease in ASLh in normal epithelia but more so in CF epithelia to reach levels well below the height of outstretched cilia required for an effective mucociliary clearance. Adapted from [78]. **B** Cell signalling mechanisms for estrogen regulation of Na^+^ absorption and Cl^−^ secretion which modulate ASLh. Estrogen signal transduction via a membrane estrogen receptor (mERα) stimulates Na^+^ absorption via transactivation of endothelial growth factor receptor EGFR, SRC kinase and matrix metalloproteases (MMP) to activate PLC and PKCδ, which in turn increase the expression and insertion of ENaC channel subunits into the apical membrane. Estrogen activation of PKCδ also phosphorylates the KCNQ1:KCNE3 K^+^ channel to cause its dissociation and reduced conductance which collapses the electrical driving force for Cl^−^ secretion via CFTR. Estrogen also reduces intracellular Ca^2+^ and thereby inhibits Cl^−^ secretion via CaCC and KCNN4. The rapid onset (< 1 min) of the effects of estrogen on sodium absorptive and chloride secretory ion transporters indicate a non-genomic mechanism of action, in addition to genomic responses on ion channel protein expression

The worsening lung function in CF and asthma in females during the reproductive years and in high estradiol states, together with the female advantage of reduced severity and mortality in COVID-19, all point to an important role for estrogen in modulating ASL dynamics in these airway diseases. Several studies which have measured ASLh directly in human bronchial epithelia have demonstrated a reduction in ASLh upon exposure to the active estrogen, 17β-estradiol, at physiological concentrations [78, 168]. Estrogen modulation of airway epithelial ion channels has been shown to be a key mechanism for the hormone effect to lower ASL height. The estrogen-induced decrease in ASLh was observed in both normal and CF bronchial epithelia, however, in CF epithelia the ASLh was greatly reduced below the lower range of the effective height of outstretched cilia (< 6 µm) (Fig. 5A).

### Estrogen regulation of ASL height and ciliary beat

The ion channel targets for estrogen to reduce ASLh have been identified as CFTR and CaCC Cl^−^ channels, ENaC and K^+^ channels (Fig. 5B). Estrogen inhibited UTP sensitive calcium entry into female CF airway epithelial cells and reduced the capacity for calcium-activated chloride ion secretion [168]. Other than a decrease in intracellular [Ca^2+^], the signalling pathway was not worked out in this latter study, although the authors eliminated estrogen effects on P2y2-R expression, IP3 signalling or de-sensitization of thapsigargin-sensitive intracellular calcium stores. While the identity of the ion channels involved in the estrogen response were not investigated, the likely candidates are CaCC (TMEM16A) channels which offer a bypass route for Cl^−^ secretion when CFTR is dysfunctional in CF airways [169]. In this way estrogen could possibly further exacerbate mucus and bacterial retention in CF airways, even if CFTR correctors or modulators are applied in female CF [170]. Another potential ion channel target is the calcium-activated KCNN4 channel located in the basolateral membranes which maintains calcium-activated Cl^−^ secretion of airway epithelial cells [171], and which may be inhibited indirectly by estrogen through the lowering of intracellular Ca^2+^ levels. Estrogen has also been show to inhibit cAMP-activated chloride secretion via CFTR [78, 172], (Fig. 5B), and to inhibit CFTR expression [173]. This is the most likely mechanism for the estrogen lowering effect on ASLh in normal non-CF bronchial epithelium (upper curve in Fig. 5A), since sodium absorption and ENaC activity are greatly reduced when wild-type CFTR is expressed in the apical membranes of airway epithelia [174]. Estrogen also inhibits cAMP CFTR mediated chloride secretion indirectly by inhibiting the activity of basolateral KCNQ1 (Kv7.1) K^+^ channels. The molecular mechanism involves non-genomic estrogen signalling via a membrane estrogen receptor (mERα) which stimulates PKCδ phosphorylation of the KCNQ1 regulatory subunit KCNE3 leading to collapse of the K^+^ conductance of the channel [175] together with estrogen-induced endocytosis of the KCNQ1 channel [176]. Under these conditions, the charge balance for Cl^−^ secretion through CFTR is lost and chloride secretion is inhibited [177]. This anti-secretory action of estrogen is female-specific and its potency varies with the stage of the estrous cycle with maximum anti-secretory responses observed at higher circulating plasma levels of estrogen during proestrus and estrus [178]. The regulation of KCNQ1 ion channels by estrogen has far-reaching consequences for sex differences in airway disease given their widespread expression in the airways and role in inflammatory lung disease [81]. Although the female sex-specific, anti-secretory, effect of estrogen on CFTR may have little consequence in healthy airways, it does pose challenges for the efficacy of CFTR modulator therapy in female CF patients for which recent clinical trials show pulmonary function outcomes less effective than in male CF patients [56]. In the CF lung, ENaC activity predominates and the action of estrogen to lower ASLh in female CF airway epithelium was shown to result from stimulation of sodium absorption via activation of ENaC [78]. The activation of ENaC drives the ASLh well below the optimal height for ciliary beating and accounts for the very low ASLh reached in high estrogen states (lower curve in Fig. 5A). Estrogen has also been shown to activate ENaC channel expression and activity in extrapulmonary epithelia, mainly kidney [179–181]. The signal transduction mechanisms mediating estrogen activation of ENaC involve estrogen binding to mERα followed by transactivation of EGFR which stimulates PKCδ-mediated insertion of ENaC channel subunits into the apical membrane [180], (Fig. 5B). It is worth noting that neutrophil elastase transactivates EGFR in CF airways [182] and ENaC is a molecular target in correcting the ASL hydration deficit in CF [127, 183–187].

**Fig. 6:**
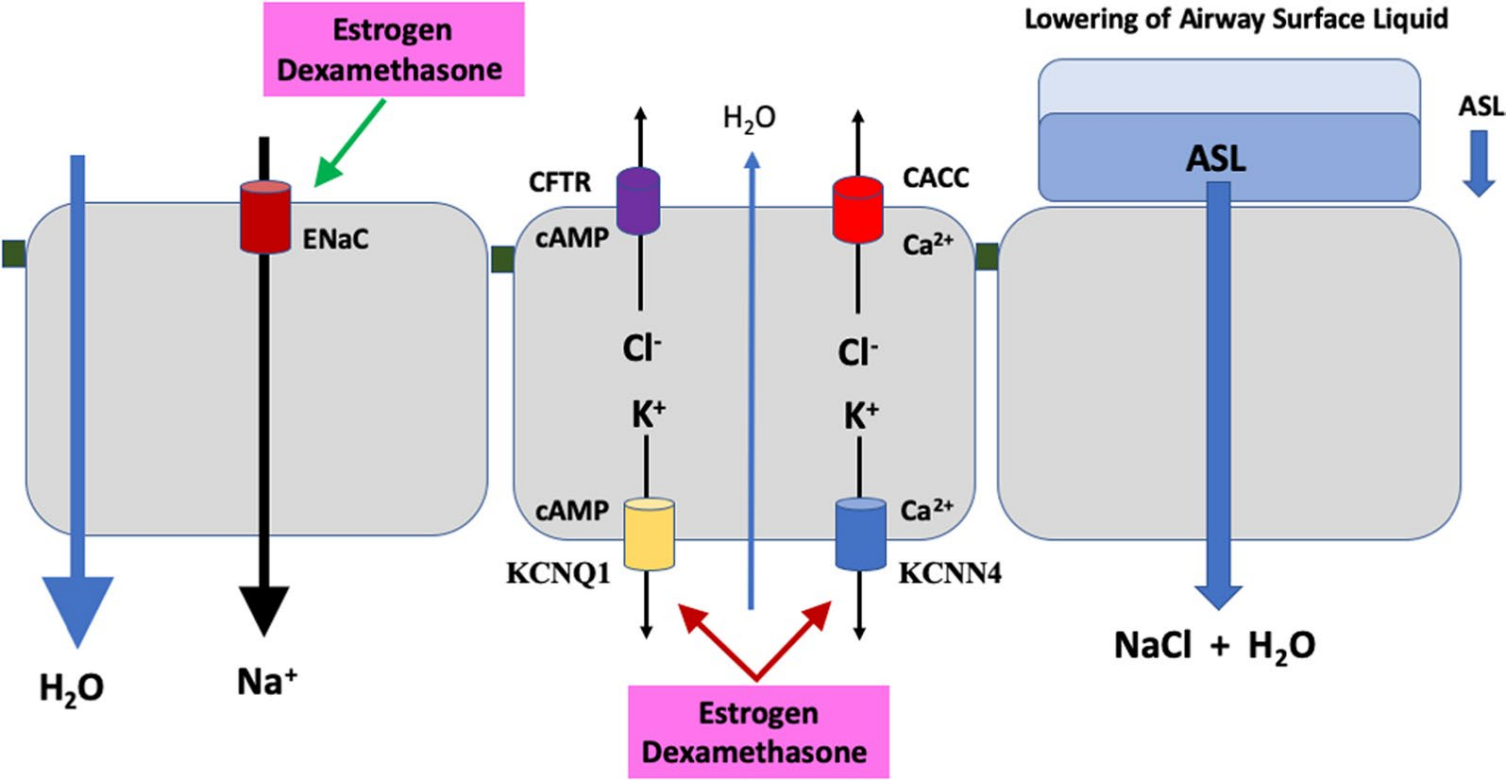
Estrogen and dexamethasone regulation of airway Na^+^ absorption, Cl^−^ secretion and ASL height. Estrogen inhibits CFTR and CaCC-mediated chloride ion secretion by inhibiting the activity of KCNQ1 and KCNN4 channels, respectively. Cl^−^ secretion activated by cAMP agonists such as forskolin or adrenergic stimulation can be rapidly inhibited by dexamethasone or estrogen acting on KCNQ1 channels. Dexamethasone inhibition of KCNN4 channels reduces the calcium-activated Cl^−^ secretion stimulated by purinergic (ATP) or muscarinergic (acetylcholine) agonists. Adapted and redrawn using data from [112, 161]. The diverse nature of the anti-secretory response indicates that estrogen and dexamethasone can reduce ASL height irrespective of the nature of chloride secretion transport pathways. Estrogen and dexamethasone can both stimulate Na^+^ absorption by increasing the expression of ENaC channel subunits into the apical membrane. The combined effects of pro-absorptive and anti-secretory actions of estrogen and dexamethasone result in increased water efflux out of the airways and lowering of ASL height which can be advantageous in reducing airway flooding in COVID-19 and alleviating morbidity and mortality in females

The effect of estrogen to lower ASLh presents a novel contributory factor to explain sex differences in CF airway disease. A combined effect of a reduction in airway secretion and stimulation of absorption would dehydrate the airway surface fluid (Fig. 6), increase mucus viscosity and reduce mucociliary clearance, which are all exacerbating factors in CF and asthma. Thus estrogen effects on ASL dynamics could contribute to sex differences in CF and asthma in which a female disadvantage is apparent. On the other hand, in respiratory diseases associated with airway flooding such as COVID-19, pneumonia, and respiratory distress syndrome, clinically proven sex differences exist with a male disadvantage in incidence and severity. The female advantage observed in COVID-19 [188] may, to a large extent, involve estrogen effects on immune responses, ACE-2R expression and a lowering of ASLh. In addition, the increased sodium absorption and decreased chloride secretion driven by estrogen should be particularly effective in lowering alveolar surface fluid volume and improve gas exchange. Such an effect would be predicted to have beneficial effects in pneumonia, respiratory disease syndrome (RDS) and COVID-19. Male sex is associated with higher incidence and severity of RDS in pre-term births [189] and estrogen has been shown to reduce respiratory distress and improve survival in preterm infants [190–192]. Estrogen receptors driving fluid absorption may be implicated in this female sex advantage. Indeed, female lung cells have been shown to express higher levels of ERβ and higher alveolar Na^+^ transport than male lung cells in vivo. Moreover, sex difference with a female advantage are also reported for adult respiratory distress syndrome [193]. Sex differences in ENaC expression have been described in adult rats where female derived rat fetal distal lung epithelial cells had higher mRNA levels of the ENaC and Na/K-ATPase [43, 189], which could be further increased by estradiol [194, 195]. Pneumonia is another respiratory disease which is characterized by alveolar flooding and shows sexual dimorphism irrespective of how the pneumonia is acquired whether in community, hospital or nursing home [196]. Pneumococcal pneumonia shows a female advantage in severity and mortality in each of these scenarios and is associated with estrogen receptor expression, further advancing the hypothesis of beneficial effects of estrogen in respiratory diseases where lung fluid hypersecretion, alveolar flooding and atelectasis (lung collapse) are characteristic clinical symptoms.

Besides an optimal ASLh, mucociliary clearance is dependent on an effective ciliary beat to propel mucus towards the throat and mouth. Estrogen is reported to either inhibit or stimulate airway ciliary beating. Direct measurements of ciliary beat intensity (CBI), the product of ciliary beat rate and amplitude, which is the most representative measure of the effectiveness of ciliary beating, showed rapid (< mins) inhibition of CBI at physiological concentrations of estradiol (10^–9^ M) in human bronchial epithelium [76]. The rapid reduction in CBI in response to estrogen indicates a non-genomic mechanism of action, possibly involving ion channels and calcium signalling [122, 197]. Other studies have reported a delayed (> 12 h) inhibition of CBF by progesterone (acting via progesterone P4 receptors) in human female tracheal airway and fallopian tube epithelium [152, 198]. Studies in murine airway showed an opposite effect of estradiol to rapidly (< secs) increase ciliary beat frequency at very low concentrations 10^–13^ to 10^–11^ M, while higher concentrations began to inhibit CBF [199].

### Estrogen regulation of ASL pH, CAPS and metabolism

Other possible targets for estrogen modulation of ASL dynamics are regulators of ASL pH, channel activating proteases (CAPs) and metabolism. The pH of ASL fluid overlying CF airway epithelia is reported to be more acidic [127, 200], however there is a caveat as these studies have been either carried out in mouse and pig models of CF, or in cultured airway CF epithelial cell lines. CF mouse models appear to be resistant to producing an acidic ASL pH in the absence of CFTR due to lack of the acidifying H^+^ pump (ATP12A), whereas the presence of this acidifying H^+^ transporter in CF pig and CF human airway epithelia may underlie their acidic ASL pH [201]. The few studies in CF patients failed to show any difference in ASL pH between non-CF and CF volunteers although the numbers of patients included in the studies was quite low [202, 203]. Although there are varying views on airway surface liquid pH, most papers show ASL to be significantly more acidic in CF than in wild-type respiratory cells. For example, a low ASL pH in CF airway models has been implicated in poor mucociliary clearance [204] along with a host of other factors, including bacterial virulence and inflammation [205], and impaired host defence [206], which could be reversed on restoring ASL pH to normal more alkaline values [136, 207]. These reports are consistent with pH-dependent CF airway defects. Such studies show abnormal acidification is related to a defect in cAMP-dependent HCO3^−^ secretion involving both CFTR and pendrin (Cl^−^/HCO_3_^−^ exchanger), concomitant with persistent H^+^ secretion by ATP12A. Moreover, small pH variations, similar to those observed between CF and non-CF airways, modulate LL-37 and hBD1 antimicrobial peptide activity. Inhibition of pendrin activity in wild-type airways recapitulates both the CF acidification and antimicrobial defect and inhibition of ATP12A activity in CF epithelia, and improves pH regulation and bacterial clearance [136, 207]. The ASL pH is regulated by several acid–base ion transporters which have been shown to be estrogen-modulable, including CFTR (acting as a HCO3^−^ channel) [207], TMEM16A (calcium-activated Cl^−^ channel), SLC26A4 anion exchanger [208], pendrin (Cl^−^/HCO3^−^ exchanger), and Na^+^-dependent bicarbonate secretion, H^+^/K^+^ adenosine triphosphatase exchange pump (ATP12A), and Na^+^/H^+^ exchangers [207]. Estrogen has been reported to activate fluid secretion in secretory epithelia by stimulating Na^+^/H^+^ exchange [209] and by activating H^+^ pumps to cause acidification [210], whereas estrogen has diverse effects on the sodium-dependent HCO_3_^−^ transporter SLC4A4 [211, 212] Cl^−^/HCO3^−^ exchange. The role of estrogen in modulating ASL pH, however, is under-investigated and presents a promising area for research in the biology of sex differences in airway disease given the role of acid–base transporters in correcting the CFTR defect [213, 214]. Irrespective of the conflicting reports of low or normal ASL pH in CF airways, an alkalinisation of ASL could be beneficial in reversing some of the hallmarks of CF lung disease, notably bacterial virulence [215], mucus viscosity, hyper-activation of ENaC and enhanced sodium absorption with concomitant lowering of ASL height [216]. The common bacterial pathogens in CF airways, PsA and *Staph aureus*, are more virulent at acidic pH, and more resistant to antibiotics as a result of biofilm production [217, 218]. These pathogenic factors can be reversed at neutral or alkaline pH [215], which can also stimulate the clearance of these pathogens aided by better hydration of mucins at higher pH values [219].

An acidic ASL pH may also cause sodium hyper-absorption through direct effects of protons to open ENaC channels and by the activation of proteases which cleave ENaC subunits to increase the open probability of the channel. Sodium absorption via ENaC exhibits pH sensitivity with extracellular acidification stimulating ENaC currents and alkalinisation reducing ENaC activity [220]. These effects of extracellular H^+^ have been attributed to acidification effects locking the ENaC channel in the open-state and preventing Na^+^ self-inhibition. Other ASL intrinsic factors may also be at play, notably pH-dependent proteolytic cleavage of ENaC by channel activating proteases (CAPs), including serine proteases prostasin, matriptase, furin, and cysteinyl proteases such as cathepsins, The activities of CAPs, which are present at excessively high levels at the apical surface of CF airway epithelial cells, are a significant component in causing Na^+^ hyperabsorption in CF airways. Estrogen has been shown to activate CAPs, both serine and cysteinyl proteases, as well as neutrophil elastase [221, 222]. Prostasin is typically inactive below pH 7.0, suggesting that it may be less relevant than cathepsins which are activated at in acidic CF airways. These CAPs show trypsin-like activity to activate ‘silent’ ENaC channels [223] with optimal pH in the range of pH 6–7, and their inhibition can reverse hyper-stimulated sodium and fluid absorption in CF airway epithelium to levels that can restore airway hydration [186]. Cathepsin proteases play a pathophysiological role in inflammatory airway diseases. In CF airways, cathepsin-S may reach ENaC in the apical membrane of epithelial cells and activate the channels even in an acidic extracellular environment [224]. Cathepsin B which is present in both normal and CF epithelia, and is secreted into ASL, has also been shown to cause Na^+^ hyperabsorption in CF airway [225]. Cathepsins B, L and S are cysteine proteases that also work at an acidic pH, and their proteolytic activity is reduced at neutral pH with the exception of Cathepsin S [226]. It should be noted that certain cathepsins are also found to be active outside of their optimal pH range of 5–6 [227]. In addition to their proteolytic activity, cathepsins, which are activated at acidic pH, can impair intrinsic ASL defence factors such as defensins [228]. In addition to activating CAPs, low ASL pH may inactivate defensins [201, 229] and impair the innate mucosal defence against pathogens in CF airways [230]. An acidic ASL has also been shown to reduce the antimicrobial activity of the ASL defensin, human β-defensin-3 (hBD-3), and the cathelicidin-related peptide, LL-37 [231]. The potential for estrogen to modulate the ion transporter regulators of ASL pH open up a new vista for research into sex differences in channel activating proteases and defensins biology in airway disease.

### Estrogen regulation of ASL pro-resolution mediators

Specialized pro-resolving lipid mediators (SPMs) such as Resolvin D1 and Lipoxin A4 are essential in promoting the termination and resolution of the body’s anti-inflammatory response to inflammation [232]. SPMs have been shown to have beneficial effects in CF airway epithelium to reduce Na^+^ absorption [233], stimulate Cl^−^ secretion [234], increase ASL height [235] as well as counteracting PsA colonization [236].

The levels of SPMs have been found to be decreased in bronchoalveolar lavage fluid sampled from CF patients compared to non-CF controls [237] and were significantly lower in female CF compared to male CF patients [238]. A bi-directional cross-talk between SPMs and estrogen signalling has been reported in several tissues [239] whereby LXA4 attenuates estrogen-induced inflammatory signalling pathways and increases the expression of ERα but decreases the expression of ERβ receptors [240]. LXA4 and estriol have structural similarity and are weak agonists of ERα in epithelial cells. LXA4, by occupying these receptors, may decrease estrogen-mediated signalling [241]. Moreover, the expression of the LXA4 receptor FPR2/ALX was shown to vary during the estrous cycle with highest expression in the proestrus phase (ovarian follicular phase) when E2 levels ae highest [242]. What this cross-talk between SPMs and estrogen means for sex differences in the resolution of airway inflammation is unknown and opens a novel avenue of research into the role of SPMs in modulating estrogen-induced inflammation, ASL dynamics and PsA virulence.

### Estrogen regulation of ASL metabolism

ASL metabolism is another potential avenue for estrogen modulation of ASL dynamics. Glucose metabolism exhibits sexual dimorphism in airway epithelium and is disrupted in inflammatory airways diseases [243, 244]. For example in CF, the airway surface liquid is characterised by high glucose concentrations [245, 246] which favour bacterial growth [247], and ASL acidification [248]. Estrogen is long known to be a key hormonal regulator of glucose metabolism [249] and modulates the expression of glucose transporters Glut1 [250], Glut2 [251], Glut4 [252] and Na^+^-coupled glucose transporters (SGLT) [253, 254] which are highly expressed in airway epithelial cells [254]. These observations present another new line of investigation into the role of estrogen-modulated glucose transporters and metabolic signalling pathways underpinning sex differences in inflammatory airway diseases.

## Synergistic actions of dexamethasone and estrogen in regulating ASL dynamics with relevance to COVID-19

Dexamethasone, a potent synthetic glucocorticoid, is widely used as an anti-inflammatory treatment in the management of airway diseases. Beyond its well-known effects on gene expression and inflammatory mediators, dexamethasone regulates the activity of potassium ion channels Kv1.3 (KCNA3) in the airway epithelium which modulate immune responses [255] as well as Kv7.1 (KCNQ1) and KCa3.1 (KCNN4) ion channels which control salt secretion and hydration of the airways [161]. The action of dexamethasone to inhibit chloride ion secretion in airway epithelium can lead to dehydration and thinning of the ASL [160, 256]. Dexamethasone inhibition of chloride secretion involves the reduction in both CFTR and CaCC Cl^−^ secretory capacity as a result of the inhibition of Kv7.1 and KCa3.1 K^+^ channels, respectively [112, 161]. The dexamethasone inhibition of Kv7.1 channels is interesting for sex differences as it involves a similar mechanism as the anti-secretory response to estrogen involving PKCδ phosphorylation of the KCNQ1:KCNE3 channel complex which causes closure of the channel, thus collapsing the electrical driving force for Cl^−^ secretion via CFTR [257]. Dexamethasone can further lower ASL height through genomic actions to stimulate sodium absorption by increasing the expression and abundance of ENaC channel subunits in the apical cell membranes of airway epithelium [159], an effect which is very similar to the molecular mechanism of action for estrogen stimulation of ENaC activity in bronchial airway epithelial cells [78]. The anti-secretory and pro-absorptive actions of dexamethasone may prove beneficial in conditions where hyper-secretion and pulmonary fluid accumulation are a feature of morbidity in COVID-19 [167]. Since estrogen regulates the expression of KCNQ1 and PKCδ [178], and ENaC channels [177], this may confer a sexual dimorphism on dexamethasone anti-secretory and pro-absorptive actions in airway epithelia. Given the common ion channel and protein kinase targets of estradiol and dexamethasone in modulating airway ASLh, it is interesting to speculate that both estrogen and glucocorticoids could synergise to reduce airway flooding and underpin the female advantage of decreased morbidity and mortality observed in COVID-19 [50]. Indeed, estrogen and dexamethasone share many common resolution targets in the airway epithelium, from reducing inflammation by repressing inflammatory cytokines and stimulating the expression of anti-inflammatory cytokines, to modulating ion and water transport and ASL dynamics to reduce airway flooding.

CF patients were considered to be at high risk of severe COVID-19 given their already compromised lung function and airway damage, however, this was not found to be the case as numerous clinical studies have shown CF patients to be spared from high mortality in COVID-19. With regards to the less bleak survival of CF patients with COVID-19 and even better survival when compared to non-CF COVID-19 patients, it appears that the high ASL viscosity and acidic pH are unfavourable for the establishment of SARS-Cov-2 infection in the airways of CF patients [134].

## Sex differences in SARS-Cov-2 infection, COVID-19 development and vaccination response.

Sex differences have been observed in various aspects of SARS-CoV-2 infection, COVID-19 development, and vaccination response. Early in the pandemic, it was noted that men were more likely to be infected with SARS-CoV-2 compared to women. This difference was observed across various age groups and geographical regions. As the pandemic evolved, sex differences in incidence became negligible, probably due to higher occupational exposure and vaccination penetration into a wider population, and enhanced immune responses in both sexes. While men and women are equally susceptible to contracting the virus, studies have consistently shown that men are at higher risk of developing severe COVID-19 illness and have a higher mortality rate compared to women. There is evidence suggesting that women tend to mount stronger immune responses to SARS-CoV-2 infection as a result of XX chromosome reinforcement of immunogenic gene expression and an enhanced immune response to estrogen. This may partly explain why women are less likely to develop severe COVID-19. The pattern of sex differences in long COVID, however, are reversed with women reporting more severe prolonged comorbidity (such as type 1 diabetes). Factors such as underlying health conditions, autoimmune susceptibility among women and behavioural differences may contribute to this discrepancy. Some studies have indicated that women may respond with better vaccine efficacy and mount a stronger immune response to COVID-19 vaccines compared to men. This heightened response may result in stronger antibody production and longer-lasting immunity.

### Sex disparities in SARS-CoV-2 infection and COVID-19 progression

Biological sex factors influence susceptibility to SARS-CoV-2 infection and the progression of COVID-19 [258, 259]. Understanding sex differences in both SARS-CoV-2 infection rates and the clinical outcomes of COVID-19 is key for developing effective prevention and treatment strategies. While females may have a lower risk of severe disease, both sexes are susceptible to infection and its consequences. Numerous studies have indicated variations in SARS-CoV-2 infection rates between males and females. While initial reports suggested similar infection rates, subsequent research has revealed a nuanced picture. Factors such as behavioural differences, hormonal influences, and immune responses [260, 261] may contribute to these variations. For instance, males tend to engage in riskier behaviours, have higher incidence of smoking and alcohol intake, potentially increasing their exposure to the virus [262]. Additionally, sex hormones, particularly estrogen, have been implicated in modulating the immune response to viral infections, potentially conferring some degree of protection to females [263]. Sex hormones, including androgens and estrogens, also impact virus entry and load through their differential effects on ACE-2 receptor expression as well as on ADAM-17 cleavage of the SARS-Cov-2 spike protein and TMPRSS2 facilitation of viral entry [86, 92, 264].

Sex disparities also manifest in the clinical outcomes of COVID-19. Studies have consistently shown that males, regardless of age, are at higher risk of severe disease, ICU admission, and mortality compared to females. The primary risk factors for severe disease are gender, age, and comorbidities including cardiovascular and metabolic diseases. Possible explanations include differences in immune response, females have a more robust T cell response and males have higher levels of innate immune cytokines [265], underlying comorbidities, and genetic factors. Male patients often present with more severe symptoms and complications, such as acute respiratory distress syndrome, thromboembolism and cytokine storm, leading to worse prognoses. Males have higher hospitalization, ICU and mortality rates than women. Cancer patients, particulalry prostate cancer, are also more prone to SARS-CoV-2 infection and are susceptible to more severe outcomes [266]. Moreover, the opposing effects of testosterone and estrogen in the RAS system discussed in section "Estrogen regulated ion channels in COVID-19" favour protective vasodilation responses to estrogen as well as decreasing the risk of coagulation and thrombosis in women with COVID-19. The presence of the double XX chromosome confers additional protective immune responses in women with the potential of duplication in the expression of immunoprotective genes [267]. Conversely, men have a less effective immune response with consequent severe clinical manifestations of the disease, together with a greater predisposition to inflammation. These sex disparities translate to a higher overall mortality in men and, in particular, in elderly men who appear to be more susceptible to severe COVID-19, likely due to a greater predisposition to infections, a weaker immune defence, and an enhanced thrombotic state compared to women [268].

### Sex differences in long COVID

As the COVID-19 pandemic continues to evolve, the phenomenon of long COVID-19 has emerged as a significant concern. Long COVID-19 refers to persistent symptoms that continue for weeks or months after the acute phase of the illness has resolved. While much attention has been focused on acute COVID-19, understanding the sex differences in long COVID-19 is essential for comprehensive patient care and management. Emerging evidence suggests that sex disparities exist in the prevalence, symptomatology, and duration of long COVID-19 [269]. While both males and females can experience long COVID-19, studies have indicated that females may be disproportionately impacted and more predisposed to developing persistent symptoms [270, 271]. Women appear to be twice as likely to develop long COVID as men, but only until around age 60 years, when the risk level becomes similar [272]. Possible explanations include differences in immune response, hormonal influences, and psychosocial factors [273, 274]. Females have been reported to experience a higher burden of autoimmune diseases [275], which may predispose them to dysregulated immune responses implicated in long COVID-19 [276]. The manifestation and severity of long COVID-19 symptoms also appear to vary between sexes. While both males and females commonly report fatigue, dyspnoea, and cognitive impairment, females may be more prone to experiencing symptoms such as headache, joint pain, and depression [277, 278]. Additionally, females with long COVID-19 may present with more severe and persistent symptoms compared to males [271]. It is important to note that no sex differences in long COVID have been observed in younger cohorts of male and female children pre-puberty [279]. This points to a role for sex hormone influences on immunomodulatory responses, in particular estrogen exacerbation of a heightened inflammatory cytokine response to SARS-Cov-2 virus particles which may have remained hidden or harboured in kidney and brain [280]. The identification of sex differences in long COVID-19 has important implications for clinical management, patient care and reproductive health [281].

### Sex differences in responses to COVID-19 vaccines

With the global rollout of COVID-19 vaccines, understanding how biological sex differences influence vaccine responses is critical for optimizing vaccination strategies and controlling the spread of the virus. Around 75% of the COVID-19 vaccinations led to reactogenicity and nearly 25% of them led to adverse effects. Major risk factors were female gender, younger age and the administration of a vaccine other than BNT162b2 [282, 283]. In a review of the literature, only 30% of COVID-19 studies broke down sex differences in vaccine effectiveness and side effects [284]. These studies have indicated variations in vaccine efficacy between males and females [285], although the magnitude and direction of these differences may vary depending on the specific vaccine platform [286, 287] as well as psychosocial influences [288]. While some vaccines have demonstrated similar efficacy rates across sexes, others have shown differential responses [289]. Early studies after the first use of mRNA-based vaccines reported women were experiencing more COVID-19 vaccine side effects than men, but it seemed to be simply the result of their body’s immune response [290–292]. While rare, women were also more likely to have anaphylactic reactions to the vaccines [293]. Other studies on sex differences in adverse events to COVID-19 vaccines have reported females responding with a higher protection rate [294–296]. Factors such as sex hormones, immune system differences, and genetic variables may contribute to these disparities. For example, females tend to mount stronger humoral immune responses to vaccination, potentially leading to higher vaccine efficacy rates compared to males. Sex-specific differences in immune responses to vaccines have been well-documented. Females generally exhibit stronger innate and adaptive immune responses, which may contribute to enhanced vaccine efficacy. Estrogen, in particular, has been implicated in modulating immune function, leading to more robust antibody production and T cell responses in females [297]. Conversely, males may be more prone to developing vaccine breakthrough infections due to weaker immune responses. Psychosocial factors may also come in to play, particularly for the gender gap in COVID-19 vaccine hesitancy which results to a large extent from women perceiving higher risks than benefits of the vaccines [298]. Understanding sex differences in vaccination impact on SARS-CoV-2 infection and COVID-19 progression has significant implications for public health interventions [299]. Tailoring vaccination strategies to account for these disparities is imperative. For instance, vaccination campaigns may need to consider prioritizing certain demographic groups based on sex-specific risk profiles [300].

## Sex differences in response to therapies in airway disease

The management of airway diseases such as CF, asthma, COPD and COVID-19 poses unique challenges, particularly due to the variability in patient responses to therapies. In recent years, there has been a growing recognition of the influence of sex bias on treatment outcomes in airway disease. Understanding these sex differences is crucial for optimizing therapeutic strategies and improving patient care.

### Sex differences in response to therapies in cystic fibrosis

Cystic fibrosis is a complex genetic disorder characterized by progressive respiratory and gastrointestinal dysfunction. Despite advances in CF research and treatment, several challenges remain in addressing sex disparities in CF therapeutic responses. Limited representation of females in clinical trials and the complexity of hormonal influences on disease pathogenesis pose significant obstacles to understanding sex-specific responses to CF therapies. Over the years, advancements in treatment modalities have significantly improved the prognosis for individuals with CF. However, the response to CF therapies, including airway clearance techniques, inhaled medications, and CFTR modulators, may vary based on sex. For instance, females with CF have been reported to exhibit greater improvements in lung function following treatment with CFTR modulators compared to males but lung exacerbations still remain higher than male counterparts even after treatment with the most advanced modulator treatments. The overall improvement of health in people with CF using transmembrane conductance regulator modulator therapy, elexacaftor/tezacaftor/ivacaftor (ETI), still shows sex-disparity in lung function and exacerbations. Pulmonary exacerbations decreased more in males (43% reduction) than females (25% reduction) [56]. Paradoxically, CFTR modulator therapy with lumacaftor/ivacaftor produced a larger sweat chloride response (a measure of CFTR correction) in women with CF than in men. This response lacked correlation with improvements in pulmonary function (FEV_1_) [301]. The basis for sex differences in CFTR correction with ETI therapy and uncoupling from pulmonary function remain unexplained but may be due to greater CFTR modulator effects on ASL dynamics and mucociliary clearance in male CF patients. The efficacy of antibiotic treatments also shows sex bias in CF patients with female patients requiring a greater number of IV antibiotic treatments than males to resolve their symptoms [54]. Our understanding of sex differences in response to therapy in CF is complicated by the multiple cellular signalling pathways modulated by estrogen in the airway epithelium which affect lung pathophysiology and outcomes [18]. For example, estrogen may suppress innate immunity in the CF lung by increasing secretory leukoprotease inhibitor (SLPI) production in the airway epithelium, resulting in the downregulation of interleukin (IL)-8 via the Toll-like receptor (TLR)–nuclear factor-κB signalling pathway [27]. The resultant inhibition of neutrophil chemotaxis could explain the poorer response to antibiotic treatments in female CF patients. Moreover, estrogen has been shown to suppress the innate immune response to respiratory *P. aeruginosa* infection [302], as well as exacerbating *P. aeruginosa* biofilm production, mucoidy [21] and virulence [76]. Such microbial virulence responses to estrogen would lead to a more resilient bacterial phenotype resistant to host and antimicrobial killing. These combined unfavourable effects of estrogen on innate immunity and bacterial infection may contribute to the sex differences to CFTR modulator therapy and antimicrobial efficacy.

### Sex differences in response to therapies in asthma and COPD

Studies have revealed notable sex differences in the response to asthma therapies. For instance, females have a higher prevalence of severe asthma and are more likely to experience exacerbations compared to males [303]. In COPD, sex differences also play a role in therapeutic responses. While smoking has historically been more prevalent among males, the incidence of COPD is increasing among females, especially in never-smokers. These observations suggest that females with COPD may experience greater dyspnoea and have a higher risk of exacerbations compared to males. Furthermore, females tend to exhibit greater bronchial hyper-responsiveness, which can impact treatment efficacy. Studies have also revealed sex differences in airway remodelling following inflammation [28], which are principally dependent on age and type of therapeutic medications.

Age-dependent sex differences in asthma can have profound effects on the efficacy of therapeutic interventions. Epidemiological data have shown that as children, boys have an increased prevalence of asthma compared to girls (11.9% vs. 7.5%, respectively) [304], and boys are also twice as likely as girls to be hospitalized for an asthma exacerbation [305].

However, during adolescence there is a decline in asthma prevalence and morbidity in males concurrent with an increase in females. By adulthood, women have increased asthma prevalence compared to men (9.6% versus 6.3%, respectively) [304, 306], and women are three times more likely than men to be hospitalized for an asthma-related event [307–309]. This increase in asthma prevalence in women compared to men is maintained until around the time of menopause, when a decrease in asthma prevalence is noted in women [310].

Additionally, hormonal fluctuations during the menstrual cycle and pregnancy can influence asthma symptoms and response to medications. The observed sex differences in asthma management have significant therapeutic implications [311, 312]. Although asthma is more prevalent in women, there are no sex-specific international recommendations for the management and prevention of asthma, and no sex-related indication for the treatment of airway diseases in general. The response to asthma therapies varies significantly based on sex, impacting treatment efficacy and disease control. Some clinical studies found that males show a greater response to inhaled corticosteroids (ICS) and bronchodilators compared to females [3, 313]. However,  an analysis of the FLAME study found no differences in response to dual bronchodilators compared to inhaled corticosteroid (ICS)/long-acting β2 agonist (LABA) in males and females [314]. A triple bronchodilator combination of ICS/LABA/LAMA was also shown to be equally effective in both men and women [315]. A small number of studies have reported superior bronchodilator responses in females compared to males with the use of combined corticosteroid and long-acting muscarinic antagonist [316]. One possible explanation for these differential ICS responses could be the interaction between estrogen and corticosteroids in the regulation of ASL dynamics via their common targeting of K^+^ ion channels and ASL dynamics as discussed in section “Synergistic actions of dexamethasone and estrogen in regulating ASL dynamics with relevance to COVID-19”. Additionally, hormonal fluctuations, particularly estrogen and testosterone levels, can influence the pharmacokinetics and pharmacodynamics of asthma medications, potentially affecting treatment outcomes.

### Sex differences in response to therapies in COVID-19

As research progresses, evidence has emerged suggesting that the response to COVID-19 therapies may differ between males and females [317]. The sex bias in vaccine efficacy is one example as discussed above. A recent study suggests that certain antiviral medications, such as remdesivir, may have differential effects in males and females [318]. Moreover, hormonal fluctuations, airway anatomy and immune system differences may impact the effectiveness of immunomodulatory therapies, such as corticosteroids and monoclonal antibodies, in male and female patients. In addition to sex differences, pregnancy affects the pathogenesis of respiratory viral infections with pregnant females often developing greater immune responses but experiencing more adverse reactions than males [319]. Despite advancements in COVID-19 research, several challenges remain in addressing sex disparities in treatment responses. Limited representation of females in clinical trials, as well as the complex interplay of biological, behavioural and psychosocial factors, pose significant challenges.

## Conclusions

**Fig. 7:**
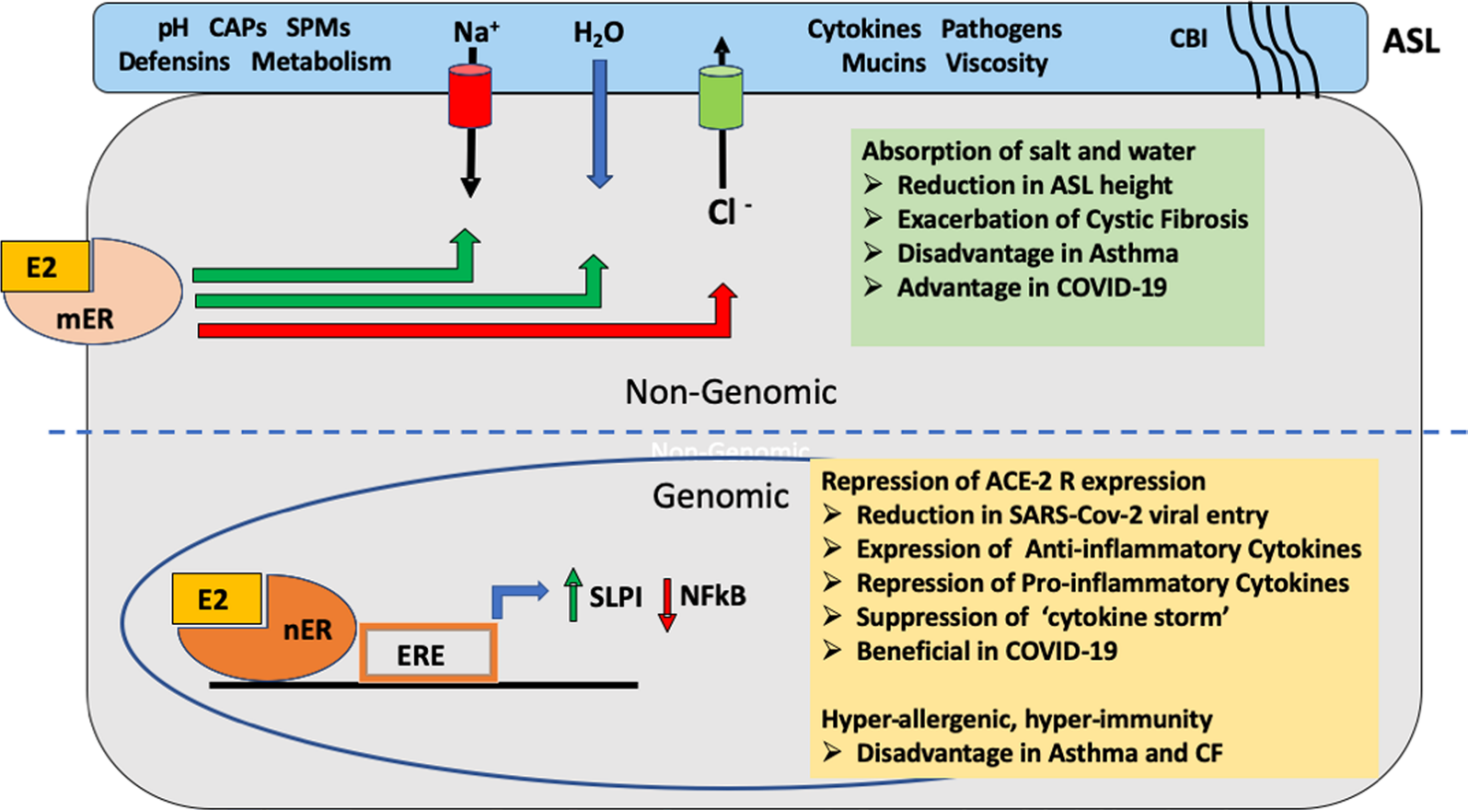
Estrogen regulated cell signalling underpinning sex differences in lung disease. Estrogen targets ion transport pathways mediating sodium absorption and chloride secretion in airway epithelial cells. Estrogen activates ENaC and stimulates Na^+^ and water flux out of the airways while inhibiting CFTR and CaCC ion channel mediated Cl^−^ secretion and water flux into the airway. These ion transport responses synergise to lower ASL height. The rapid action of estrogen on ion channel activity indicate membrane-initiated ‘non-genomic’ signal transduction via membrane estrogen receptors such as mERα. Although the pro-absorptive and anti-secretory responses to estrogen are rapid, they are also long-lasting, most likely as a result of sustained protein kinase phosphorylation of the ion channels and delayed genomic activation of mRNA expression of ion channel subunits. Estrogen effects on ASLh may also synergise with the repression of pro-inflammatory cytokines and anti-inflammatory/pro-resolution actions which are transduced by genomic signalling via nuclear estrogen receptors.

Sex differences in airway diseases involving sex steroid hormones are well-established in both basic research and clinical studies [320]. The underlying cellular and molecular mechanisms are myriad and complex. In this review we focused on the role of estrogen in regulating ion channels and airway surface liquid dynamics which appear to span a wide range of airway diseases including cystic fibrosis, asthma and COVID-19. Estrogen actions on ASL dynamics underpin sex differences in airway disease in combination with the hormone effects to activate the protective arm of RAS and reinforce innate immunity and anti-inflammatory responses (Fig. 7). At this juncture, it is important to understand that sex differences may be due not only to estrogen signalling but also to differences in expression patterns of estrogen receptor subtypes and their sensitivity to circulating estradiol [321]. Sex-dependent comorbidities may negatively influence biological sex advantages for females as has recently been described in COVID-19 [322]. Moreover, airway diseases in which females would be predicted to have an advantage such as in COPD because of lower incidence of tobacco smoking are showing a higher incidence in women who do not smoke, possibly due to estrogen sensitization of inflammatory responses to passive smoking [323].

Estrogen-regulated ASL dynamics, in particular ion channels, plays a fundamental role in the modulation of ASL height and mucociliary clearance, which are essential for maintaining healthy respiratory function. Dysregulation of ion channels leads to altered ASL electrolyte and water composition, viscosity and volume, contributing to various respiratory diseases and conditions. Estrogen effects on ASL dynamics highlight the hormonal influence beyond reproductive functions. Modulation of ENaC, CFTR, CaCC, KCNQ1 and KCNN4 ion channels by estrogen negatively affect airway surface liquid height and mucociliary clearance which impact on mucus production, bacterial virulence and airway inflammation, contributing to CF and asthma pathophysiology. In contrast, estrogen has a beneficial role in airway diseases characterised by pulmonary edema and alveolar flooding such as pneumonia, ARDS and COVID-19. The actions of estrogen to reduce airway fluid secretion combined with suppression of the expression of both pro-inflammatory cytokines and ACE2 receptors may contribute to the female sex advantage seen in COVID-19 and other forms of acute respiratory distress syndrome. Glucocorticoids used to treat a wide range of inflammatory airway diseases for their anti-inflammatory effects possess additional therapeutic targets such as ion channels to reduce ASL volume and help resolve pulmonary edema in severe COVID-19. As many of the ion channels targeted by dexamethasone are also modulated by estrogen, a synergistic action of estrogen and glucocorticoids may confer additional benefits for a female advantage in COVID-19 and ARDS.

## Perspectives and significance

Understanding the effects of estrogen on ion channels and ASL dynamics in airway disease holds significant therapeutic potential. Estrogen modulation of ion channels and ASL dynamics underpins sex-related differences in airway disease prevalence, symptomatology, and treatment response. Targeting specific ion channels modulated by estrogen could offer novel therapeutic avenues for the management of CF, asthma, COVID-19, COPD and ARDS, potentially leading to more effective and personalized treatments. Further investigation into the precise molecular mechanisms underlying estrogen and ion channel interactions is warranted to unlock the full therapeutic potential of targeting estrogen-related pathways for the management of airway disease. Longitudinal studies assessing clinical treatment, pharmacological and vaccine effectiveness across sexes are warranted to guide ongoing therapy efforts. Additionally, exploring the role of sex hormones in treatment responses may provide insights into novel approaches for improving drug efficacy and reducing adverse events in women.

## Data Availability

Links to sources of all data and material used in this article are given in the text. Any additional source data and material are available on request from the authors.
